# Gamification-based tele-rehabilitation for physical therapy in patients with Parkinson’s disease: A scoping review

**DOI:** 10.1371/journal.pone.0326705

**Published:** 2025-08-29

**Authors:** Somayeh Norouzi-Ghazbi, Shaghayegh Mirbaha, Zoe Li, Jan Andrysek, Roger Goldstein, Sander L. Hitzig

**Affiliations:** 1 Department of Occupational Science and Occupational Therapy, Temerty Faculty of Medicine, University of Toronto, Toronto, Ontario, Canada; 2 St. John’s Rehab Research Program, Sunnybrook Research Institute, Sunnybrook Health Sciences Centre, Toronto, Ontario, Canada; 3 Institute of Biomedical Engineering, University of Toronto, Toronto, Ontario, Canada; 4 Holland Bloorview Kids Rehabilitation Hospital, Toronto, Ontario, Canada; 5 Departments of Medicine and Physical Therapy, Temerty Faculty of Medicine, University of Toronto, Toronto, Ontario, Canada; 6 West Park Healthcare Centre, University Health Network, Toronto, Ontario, Canada; 7 Rehabilitation Sciences Institute, Temerty Faculty of Medicine, University of Toronto, Toronto, Ontario, Canada; Ziauddin University, PAKISTAN

## Abstract

**Objective:**

A scoping review was conducted to explore what is known about gamification-based tele-rehabilitation (GBT) to enable physical therapy in home settings for people with Parkinson’s disease (PD).

**Methods:**

The peer-reviewed literature (OVID Medline, OVID EMBASE, CINAHL EBSCO, and Scopus databases) was searched from January 2010 to May 2024, and 24 articles met the inclusion criteria. The methodological quality of the studies was assessed using the Downs and Black evaluation tool, and levels of evidence were assigned using a modified Sackett scale.

**Results:**

The majority of the 24 studies were of poor to fair methodological quality (83%), and all used a quantitative design with small sample sizes. The focus of the included studies was to enhance whole-body rehabilitation, with most addressing the upper extremities. Thirteen studies customized their games, whereas others utilized strictly commercial systems (n = 11). Eight studies reported no adverse events while the rest did not report on these. Eight studies indicated that participants maintained high levels of motivation and adherence in home settings.

**Conclusion:**

GBT has the potential to offer a safe, engaging and effective physical therapy to the PD population in home settings, but further research is warranted to help delineate clearer implementation considerations.

## Introduction

Parkinson’s disease (PD) is a progressive brain disorder that affects the nervous system [1]. It occurs when the brain cells responsible for making dopamine, a chemical that coordinates movement, breakdown or die. PD is best known for its motor symptoms, which include increased muscle tone (stiffness), slow movement (bradykinesia), resting tremor, and impaired walking balance, and posture. Worldwide, there are more than six million people with PD, which represents almost a three-times increase compared to the 2.5 million with PD in 1990 [2]. PD is typically managed by medications (i.e., L-DOPA and dopamine agonists); however, the disease remains progressive, and the therapeutic window narrows after a few years, which consequently results in patients experiencing diminished returns and reduced functioning [3]. To help optimize physical capacity, a multidisciplinary approach, including physical therapy is recommended for PD patients [4]. A recent meta-analysis has shown that physical therapy interventions are highly effective in managing motor symptoms, and improving balance, gait and functional mobility for persons with PD [5,6].

A crucial component of physical therapy is home exercises, which often require patients to complete a set of repetitive exercises in between their appointments with their therapists to help maintain therapeutic gains. However, there is low adherence to physical therapy home-prescribed exercise programs, which may stem from patients having poor motivation due to low self-efficacy, depression or anxiety, a sense of helplessness, increased pain, and poor social support [7]. Studies that examined adherence to physical therapy have found that non-adherence is as high as 70% [8], especially in the case of home-based and unsupervised programs [9,10]. Several detrimental clinical outcomes have been associated with non-adherence to physical therapy home-based exercises, including worsening symptoms, reduced functional ability, and exacerbation of chronic conditions. This can lead to increased recovery time; adding expense to the client and health system [7].

A promising approach to increase patient motivation and satisfaction with physical therapy is to pair it with tele-rehabilitation [11]. Tele-rehabilitation is a sub-discipline of telemedicine that integrates rehabilitation and telehealth technologies, such as videoconferencing, educational patient portals, and wearable sensors, among others [12]. In particular, game-based tele-rehabilitation (GBT) solutions may be particularly motivating for patients as they introduce challenge, engagement, and rewards; making physical therapy more enjoyable and goal-driven [13]. Game-based solutions can be categorized as either serious games or those that incorporate gamification elements.

Sailer et al. [14] define gamification as the use of game design elements in non-game contexts to encourage a desired type of behavior. Within this context, games are employed to eliminate mental barriers (e.g., fear) to undertaking physical activities. Game design elements may also include reward collection, encouraging reminders, a to-do list of the daily exercises, feedback, or it may rely on attractive visualizations of task activities such as introducing a virtual exercise coach or video demonstration of the exercises. The presence of challenges with reward and fail options in serious games can foster higher levels of engagement and motivation while also enabling an automatic assessment of the correct implementation of the target exercises [13].

Despite the growing interest in GBT, the existing literature on game-based rehabilitation for PD primarily focuses on virtual reality (VR) as an intervention, with limited emphasis on tele-rehabilitation as a standalone approach. Several reviews [15–17] have examined the effects of VR on gait, balance, and manual dexterity in PD patients. However, the reviews did not include studies that incorporated tele-rehabilitation as a key component or were not focused on home-setting solutions. While other reviews [18,19] included studies that used a tele-rehabilitation element alongside VR, the focus was on multiple conditions, such as multiple sclerosis and stroke, in addition to PD. Therefore, the objective of the present scoping review was to provide a descriptive overview of the existing literature on tele-rehabilitation solutions for physical therapy in PD patients in home settings, focusing specifically on those incorporating gamification elements, such as reward collection, interactivity, engagement, and brain stimulation to boost motivation. The intended aim was to better understand the intersection of gamification and tele-rehabilitation while also identifying insights on how to best implement GBT to meet the physical therapy needs of the PD population in their homes.

## Materials and methods

We followed the five-step scoping review framework proposed by Arksey & O’Malley [20] and the subsequent revisions introduced by Levac, Colquhoun and O’Brien [21]. This included (1) identifying the research questions; (2) identifying relevant studies; (3) screening and selecting studies; (4) charting the data; and (5) collating, summarizing, and synthesizing the results. The optional step of consulting with stakeholders was not undertaken for the present review. The protocol for this review was registered in OSF (Open Science Framework – https://osf.io/a7xfg). We also referred to the Preferred Reporting Items for Systematic Reviews and Meta-analyses extension for Scoping Reviews Checklist (PRISMA-ScR) (**see** S1 Appendix **for the completed PRISMA-ScR checklist**) [22].

### Research question

The research question guiding this scoping review was: what is known about GBT to enable physical therapy in home settings for people with PD? Through this review, we aimed to: (1) explore the key features of technology systems that have been used with the PD population; (2) describe their effectiveness for PD patients; and (3) identify gaps and challenges in the adoption of GBT physical therapy solutions for PD. As noted, no studies have focused on the implementation considerations of using GBT paired with physical therapy with the PD population. Mapping out the different technologies, approaches, and end-user experiences is critical for informing clinical practice and for supporting the design of future studies. Hence, a scoping review centered on implementation considerations can offer valuable insights regarding the feasibility of utilizing GBT physical therapy-based approaches in home settings for individuals with PD, and for identifying strategies to optimize their adoption by both patients and healthcare providers.

### Information source and search strategy

As the topic is related to both the medical field and engineering, relevant studies were identified through the searching of four major electronic databases (OVID Medline, OVID EMBASE, CINAHL EBSCO, and Scopus) between January 2010 and May 2024 In order to retrieve information from the electronic databases, all possible combinations of the following key word strings were used: Game OR gamification OR user-computer interface OR software, mobile OR cellphone OR iOS OR Android, app OR application OR program, Rehabilitation OR Physiotherapy OR Physical therapy, Tele OR Remote OR Virtual OR home exercises OR Unsupervised. An expert librarian supervised the development of the search strategy. Details of the search terms and search strategies in all four databases are shown in S2 Appendix. Our results from each database were exported to a systematic review software system, Covidence, which automatically de-duplicated the extracted data [23].

### Inclusion and exclusion criteria

The inclusion criteria were studies of any design (quantitative, qualitative), published in English with adult PD individuals (18 years old and older), and that developed and/or evaluated technologies (software and/or hardware) to facilitate home-based physical therapy exercises. As well, the study needed to incorporate a gamification element. To ensure alignment with current standards in the rapidly evolving technological landscape, our inclusion criteria focused on studies published from 2010 onwards.

We excluded (1) book chapters, conference papers, commentaries, gray literature, review papers, mini-reviews, and letters; (2) studies that did not include gamification features/elements (e.g., reward collection, interactive, engaging, fun, brain stimulation to boost motivation); (3) immersive-VR; and (4) studies that only presented the conceptual designs of their systems (i.e., no PD patient involvement or no device development).

### Study selection process

The selection of studies was conducted in two phases: (1) screening of titles and abstracts and (2) full-text screening. Both phases were conducted independently by three authors (SNG, SM, ZL), who undertook an inter-rater screen of the first 80 abstracts (Phase I) and the first 30 full-text articles (Phase II) to test their rates of agreement. An agreement of 80% was set as the target, which was obtained in the title and abstract screening and full-text review phases. Any disagreements were discussed in consultation with a pre-identified fourth reviewer (SLH).

### Data extraction

A review of a subset of articles was conducted to ensure the accuracy of data extracted by the authors (SNG, SM, ZL). The remaining articles were then divided across the three reviewers for data extraction. The extracted data encompassed study purpose and design, features of the software and hardware systems (i.e., game system, sensors, functional features, etc.), characteristics of the patient population (i.e., age, sex, etc.), intervention (exercise programs, type of exercises, etc.), adverse effects associated with using the technologies, and study outcomes.

After reviewing the abstracts and identifying papers for inclusion, three reviewers (ZL, MA, SS) conducted a quality assessment of each relevant article using the Downs and Black (D&B) tool [24]. Any disagreements in scoring were resolved through consultation with the fourth author (SM). This tool is comprised of 27 questions designed to assess external and internal validity, with a focus on evaluating bias and confounding factors. The last question in the tool, originally scored on a 0–5 scale, was adjusted to a 0–1 scale. A score of 1 was given if the study included a power or sample size calculation, while a score of 0 was assigned if neither was provided nor any explanation on the appropriateness of the sample size. Consequently, the maximum score any reviewed article could achieve was 28, with a higher score reflecting higher methodological quality. The Sackett Scale was then used to assign a level of evidence to each study (**See Table 1**), with level 1 representing the highest level and level 5 representing the lowest [25].

**Table 1:** Sackett Scale level of evidence.

Level of Evidence	Type of Study
1A	Systematic reviews of randomized controlled trials
1B	Individual randomized controlled trials with narrow confidence interval
2A	Systematic reviews of cohort studies
2B	Individual cohort studies and low-quality randomized controlled trials
3A	Systematic reviews of case-control studies
3B	Case-controlled studies
4	Case series and poor-quality cohort and case-control studies
5	Expert opinion

## Results

**Fig 1:**
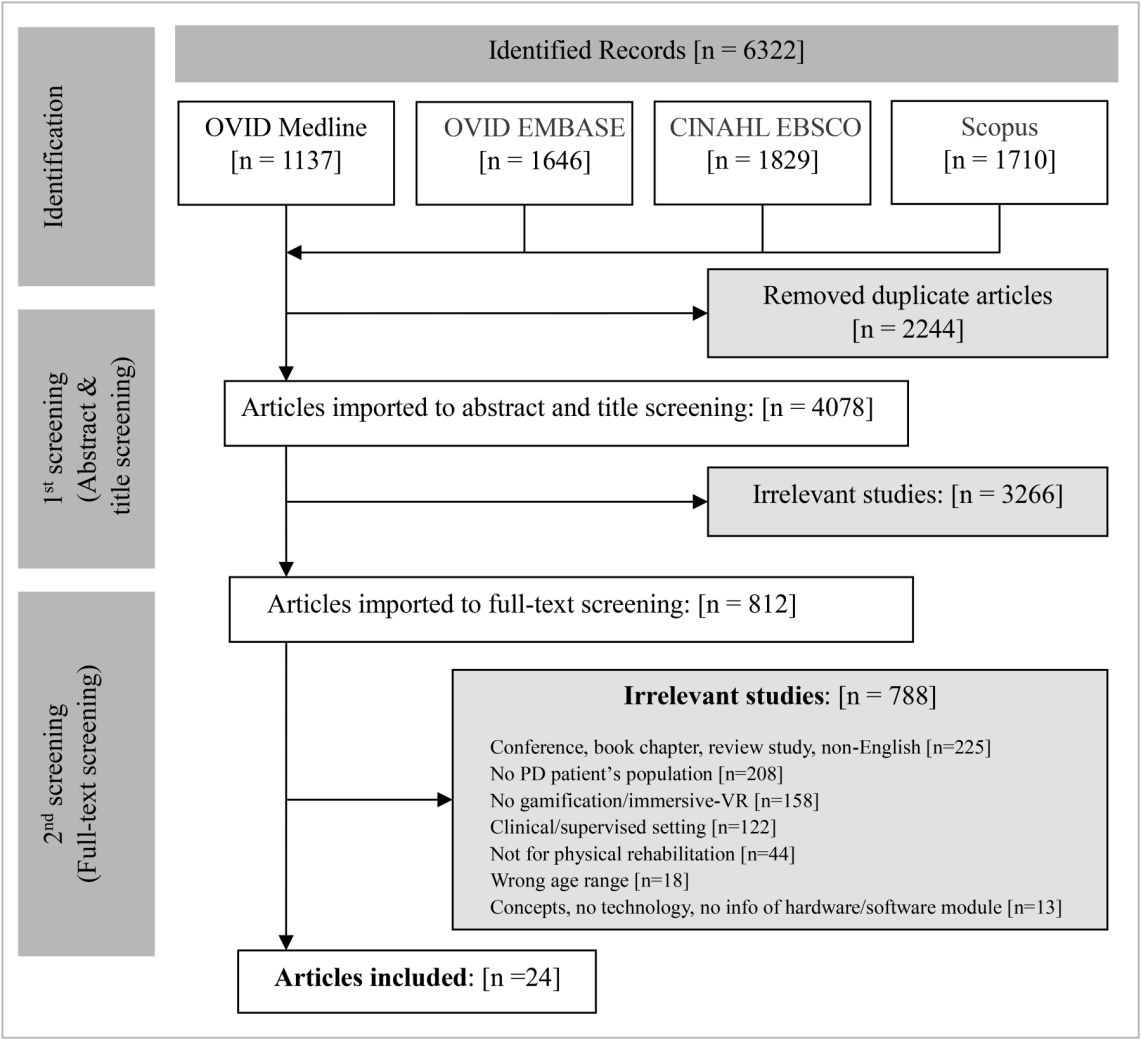
PRISMA (Preferred reporting items for systematic reviews and meta-analysis). Reporting checklist.

The search strategy yielded 6,322 abstracts. Following de-duplication, 4,078 articles remained, and 812 full texts were subsequently screened. Twenty-four articles met the inclusion criteria. The PRIMSA flowchart is shown in **Fig 1**.

The mean D&B score (**summarized in Table 2**) was 16.3, and scores ranged from 11 to 24 out of a possible total score of 28, with the majority being regarded as having poor (n = 7) [26–32] or fair (n = 12) [33–44] methodological quality (see Table 2). Only five [45–49] were of good methodological quality. After assigning the Sackett levels of evidence, three articles were level 1b, 4 articles were level 2b, 7 were level 3b, and 9 were level 4.

**Table 2:** Study bias assessment and assigned level of evidence.

Author	D&B Score	Level of Evidence	Author	D&B Score	Level of Evidence
Albiol-Perez [26]	11	2b	Gandolfi [46]	24	1b
Amprino [27]	14	3b	Hashemi [40]	19	2b
Bacha [33]	15	3b	Herz [41]	17	3b
Barth [45]	20	1b	Holmes [42]	17	2b
Brachman [35]	18	2b	Negrini [47]	20	3b
Blanc [34]	18	3b	Nuic [48]	20	1b
Cikajlo [28]	11	4	Nuic [30]	14	4
Cikajlo [36]	15	3b	Palacios-N. [31]	11	4
Cikajlo [37]	17	4	Pelosin [32]	11	4
Dauvergne [29]	12	4	Cornejo Th. [38]	15	4
Del Pino [49]	24	2b	Vallabhajosula [43]	15	4
Esculier [39]	18	4	Zeigelboim [44]	15	3b

### Study characteristics

All 24 studies used a quantitative methodology. This included: five clinical studies [35,36,40–42], three observational clinical studies [26,27,34], five pilot clinical studies [30–32,39,49], one non-randomized pilot study [37], three single-blind randomized controlled trials (RCT) [45,46,48], three pre-post observational studies [28,29,33], one cross-sectional study [44], two case studies [38,43] and one comparative study [47]. These studies were conducted across eight countries, with the majority (66%) done in Europe. The breakdown of countries was: France (*n = *4) [29,30,34,48], Spain (*n = *3) [26,31,49], Italy (*n = *4) [27,32,46,47], Poland (*n = *1) [35], Slovenia (*n = *3) [28,36,37], Brazil (*n = *2) [33,44], Canada (*n = *2) [39,42], The United States *(n = *2) [41,43], Iran (*n = *1) [40], Germany (*n = *1) [45], and Israel (*n = *1) [38].

### Participant characteristics

Across all studies (**see Table 3**), the sample size ranged from 2 to 76, with an average of 20 participants. All studies reported participants’ ages, with a mean age range between 46 and 80 years of age. Twenty studies reported on participants’ disease severity using the Hoehn and Yahr (H&Y) scale [50], where classifications ranged between I and IV, and the majority (73%) were classified in stages II-III. Only one study used the Unified Parkinson’s Disease Rating Scale (UPDRS) [51] to report on the disease severity, while twelve studies used both H&Y and the UPDRS to characterize severity. The H&Y stage-range marks the transition from bilateral symptoms without balance impairment (Stage II) to the onset of postural instability with preserved physical independence (Stage III). Similarly, mean UPDRS III and Movement Disorder Society-UPDRS (MDS-UPDRS) III scores largely fell between approximately 27 and 34, except for one study that had a score as high as 51 [46], which suggested that participants generally exhibited mild to moderate motor symptom severity. Cognitive status was assessed in 16 studies using the Mini-Mental State Examination (MMSE) [52] or Montreal Cognitive Assessment (MoCA) [53], with mean MoCA scores ranging from 25.8 (standard deviation [SD]=2.8) to 26.77 (SD = 1.48), indicating intact or mildly impaired cognition. Twelve studies excluded participants with severe cognitive impairments (i.e., MMSE < 24).

**Table 3:** Intervention and patient population characteristics.

Author	Participants Characteristics					Targeted Body Part	
	Mean Age (SD) or Range	Sample Size	Sex (M/F)	Disease Severity (stage range or stage/ score Mean (SD)	Cognitive Status	Tele-rehabilitation Program	
Albiol-Perez [26]	M = 79.6 (SD = 5.8)	10	4/6	NR	MMSE > 24/30	WB	**Exercise**: Balance, weight-transfers. **Schedule**: 1 hour/session, 2-3x/week, 15 sessions.
Amprino [27]	M = 71.1 (SD = 9.2)	20	11/9	**H&Y**: M = 2.2 (SD = 0.9) **UPDRS III**: M = 33.7 (SD = 5.9)	MMSE > 27/30	WB	**Exercise:** Leg agility, postural stability, gait, lateral and frontal weightlifting of upper limbs. **Schedule:** 1 hour/session.
Bacha [33]	M = 63.4 (SD = 5.7)	14	10/4	**H&Y**: I-III	MMSE > 22/30	WB	**Exercise:** Plugs the cracks using hands and feet, popping bubbles with hands, stepping left or right jumping to steer a raft. **Schedule:** 1 hour/session, 2x/week for 7 weeks.
Barth [45]	M = 73.56 (SD = 9.28)	25	15/10	**H&Y**: I-IV **UPDRS III**: CG: M = 31.0 (SD = 16.5) EG1:M = 26.1 (SD = 14.8) EG2:M = 27.9 (SD = 14.6)	Individuals without severe dementia	WB	**Exercise:** Upper limb-abduction and elevation, lower limb-hip and knee extension. **Schedule:** 2x/week for 3 weeks.
Brachman [35]	CG: M = 65.3 (SD = 9.2) EG: M = 69.5 (SD = 7.2)	CG: 12 EG: 12	CG: 7/5 EG: 8/4	**H&Y**: II-III **UPDRS III**: M = 31.7 (SD = 10.3)	MMSE > 24/30	WB	**Exercise:** Balance, leaning, reaching, trunk rotations, and alternating steps. **Schedule:** 3x/week for 4 weeks.
Blanc [34]	M = 69 (SD = 8)	31	17/14	**H&Y**: I-IV M = 1.86 (SD = 0.86)	NR	WB	**Exercise:** NR **Schedule:** For 1 year
Cikajlo [28]	M = 76 (SD = 7)	NR	NR	**H&Y**: II-III **MDS-UPDRS III**: Pre: M = 29.5(SD = 10.3) Post: M = 27.3(SD = 10.4)	NR	UL	**Exercise:** Hand exercise, fruit picking. **Schedule:** 30 minutes/session, 10 sessions over 3 weeks.
Cikajlo [36]	M = 68 (SD = 7)	NR	12/16	**H&Y**: II-III **UPDRS III**: M = 29.5 (SD = 10.3)	MMSE > 24/30	UL	**Exercise**: Fruit picking game. **Schedule**: 30 minutes/session, 10 sessions over 3 weeks.
Cikajlo [37]	HG: M = 62.3 (SD = 7.3) OG: M = 69.5 (SD = 5.8)	HG: 7 OG: 21	HG: 3/4 OG: 9/12	**H&Y**: II-III	NR	UL	**Exercise**: Hand exergames. **Schedule**: 30 minutes/session, 10 sessions over 2–3 weeks.
Cornejo Thumm [38]	M = 56.5 (SD = 14.8)	2	1/1	**H&Y**: III **MDS-UPDRS III**: M = 29.5 (SD = 0.7)	NR	WB	**Exercise:** Negotiating obstacles on an obstacle-lined pathway. **Schedule:** 45 minutes/session, weekly training for 12 months.
Dauvergne [29]	M = 65 (SD = 7.3)	16	12/4	**H&Y**: II-III **MDS-UPDRS**: M = 31.4 (SD = 15.9)	MMSE > 24/30	Hand	**Exercise**: Rhythmic skills (finger tapping). **Schedule**: 30 minutes/session, 3x/week for 6 weeks.
Del Pino [49]	M = 66.4 (SD = 8.5)	20	10/10	**H&Y**: II-III	MoCA EG: Pre: M = 23.6 (SD = 2.7) Post: M = 25.4 (SD = 2.7) CG: Pre: M = 23.5 (SD = 3.4) Post: M = 24.1 (SD = 2.9)	WB	**Exercise:** Cognitive and motor skills training via vCare system using virtual coach & serious games. **Schedule:** 30 minutes/session, 5x/week for 4 months.
Esculier [39]	M = 61.9 (SD = 11)	11	6/5	**UPDRS III**: M = 18.4 (SD = 5.4)	MMSE > 27/30	WB	**Exercise:** Balance, Table Tilt, Ski Slalom, Balance Bubble, Ski Jump, Penguin Slide. **Schedule:** 40 minutes/session, 3x/week for 6 weeks.
Gandolfi [46]	M = 67.4 (SD = 7.2)	EG: 38 CG: 38	EG: 23/15 CG: 28/10	**H&Y**: II-III; **UPDRS III**: EG:M = 50.8 (SD = 24.1) CG:M = 44.1 (SD = 24.0)	MMSE > 24/30	WB	**Exercise**: Balance, weight shifting, foot stepping, controlled movements. **Schedule**: 50 minutes/session, 3x/week for 7 weeks.
Hashemi [40]	M = 60.1 (SD = 7.3)	CG: 15 Supervised VR: 15 Unsupervised VR: 15	CG: 12/3 Supervised VR: 9/6 Unsupervised VR: 8/7	**H&Y**: I-IV	MMSE > 24/30	UL	**Exercise**: Hand games (reaching, tracking, rotating wheel). **Schedule**: 75 minutes/session, 3x/week for 24 sessions.
Herz [41]	M = 66.7 (SD = 7.2)	11	6/5	**H&Y**: II	MMSE > 25/30	WB	**Exercise:** Wii-hab, tennis, bowling, boxing. **Schedule:** 1 hour/session, 3x/week for 4 weeks.
Holmes [42]	M = 66.6 (SD = 5.6)	11	7/4	**H&Y**: M = 2.3 (SD = 0.4) **UPDRS III:** M = 25.3 (SD = 11.9)	Cognitively intact	WB	**Exercise**: Balance board games. **Schedule**: 30 minutes/session, 3x/week for 12 weeks.
Negrini [47]	G1: M = 67.0 (SD = 9) G2: M = 66 (SD = 8)	G1: 11 G2: 16	G1: 5/6 G2:9/7	**H&Y**: II	MMSE > 26/30	WB	**Exercise:** Table Tilt, Ski Slalom, Balance Bubble, Ski Jump, Penguin Slide. **Schedule:** 20 minutes of using Wii Fit games, 2x/week for 5 weeks or 3x/week for 5 weeks.
Nuic [48]	EG: M = 68.6 (6.9) CG: M = 64.8 (8.2)	25	14/11	**H&Y**: III-IV **UPDRS III**: M = 30.2 (SD = 14.0)	MMSE > 24/30	WB	**Exercise:** Foot lifting and postural control, coin collecting and avoiding obstacles, perform lateral displacements, knee flexion/extension, trunk rotations and arm movements. **Schedule:** 18 sessions over 6 weeks.
Nuic [30]	M = 64.0 (SD = 5.8)	10	5/5	**H&Y**: I-III M = 3.3 (SD = 0.5) **UPDRS III**: M = 20.3 (SD = 7.8)	MMSE > 24/30	WB	**Exercise**: Rapid movements of limbs, pelvis & trunk with lateral, vertical & forward displacements. **Schedule**: 18 sessions over 6 weeks.
Palacios-Navarro [31]	M = 67 (SD = 3)	7	4/3	NR	NR	LL	**Exercise**: Lateral leg movements. **Schedule**: 4x/week for 5 weeks, 10 hours of treatment.
Pelosin [32]	M = 63.2 (SD = 9.3)	10	6/4	**H&Y**: I-III, **UPDRS III**: M = 18.7 (SD = 5.4)	MMSE > 24/30	WB	**Exercise:** NR. **Schedule:** 30 minutes every day for 1 week.
Vallabhajosula [43]	M = 69 (SD = N/A)	1	1/0	**H&Y**: III	MoCA > 24/30	WB	**Exercise:** Stepping, trunk rotation, balance challenges. **Schedule:** 1hour/session, 3x/week for 8 weeks.
Zeigelboim [44]	M = 57.6 (SD = 18.7)	16	10/6	NR	NR	WB	**Exercise**: Vestibulo-visual interactions, static and dynamic postural stability. **Schedule**: 50 minutes/session, 2x/week.

EG: Experimental Group, G1: Group 1, G2: Group 2, HG: Home Group, H&Y: Hoehn & Yahr Scale, LL: Lower Limb, M: Mean, MoCA: Montreal Cognitive Assessment, MMSE: Mini-Mental State Exam, N/A: Not Applicable, NR: Not Reported, OG: Outpatient Group, SD: Standard Deviation, UPDRS: Unified Parkinson’s Disease Rating Scale, UL: Upper Limb, VR: Virtual Reality, WB: Whole-Body.

### Rehabilitation process and outcomes

Eighteen articles [4 level 2b, 6 level 3b, 5 level 4, and 3 level 1b] focused on whole-body rehabilitation that predominantly involved balance-focused exercises with controlled lower extremity and trunk/postural movements (weight shifting or bearing, stepping, leaning etc.) prompted by the VR technology [26,27,30,32–35,38,39,41–49]. All 18 of these studies aimed to treat gait and balance disorders, and to evaluate improvements in postural stability, independence, confidence, as well as perceived balance in performing activities of daily living in PD patients. Except for one study [34], all the studies that targeted whole-body rehabilitation reported intervention durations ranging from several weeks to a year [26,27,30,32,33,35,38,39,41–49]. The planned frequency of the sessions ranged from every day to four times per week and they were scheduled as 30- to 75-minute long sessions [26,27,30,32–35,38,39,41–48].

The most commonly employed outcome measures used in these studies to assess functional mobility, postural stability and perceived balance were the Berg Balance Scale (BBS) [54], MDS-UPDRS [51], the Tinetti Performance-Oriented Mobility Assessment (POMA) [55], the Activities-Specific Balance Confidence (ABC) Scale [56], and the United Theory of Acceptance and Use of Technology (UTAUT) questionnaire [57]. All 18 studies demonstrated positive outcomes in terms of improved balance, balance confidence, gait, motor function, mood, and acceptance rates among persons with PD [26,27,30,32–35,38,39,41–49].

In addition to physical outcomes, the study by Del Pino and colleagues [49] undertook a cost-utility analysis comparing their GBT solution (n = 10) to a control group of patients (n = 10) who underwent standard clinical care. They found that the cost of GBT was €2243.07 while standard care cost €5108.26 over a 3 month period. The authors attributed the cost savings to not requiring the physical presence of the professional for the GBT participants. Similarly, Gandolfi et al. [46] examined costs between their GBT participants who underwent a TeleWii intervention (n = 38) versus those who participated in an in-clinic sensory integration balance training (SIBT; n = 38) program, which found the total treatment cost was €246.75 per patient for the GBT group and €493.50 per patient for the SIBT group. When taking into account indirect costs as well as equipment costs, the total cost for GBT was €383.55 per patient for the TeleWii group and €602.1 per patient for the SIBT group. One case study [38] using a customizable game estimated their system would cost approximately $2,000 (currency not specified). Other included studies highlighted the cost-effectiveness of GBT compared to traditional therapy (e.g., Negrini et al. [47]) but did not provide data to support these claims or did not discuss costs at all.

Five studies targeted upper-limb rehabilitation [1 level 2b, 1 level 3b, and 3 level 4], and all of these specifically focused on exergames that involved rotating and picking up objects with the hands and arms [28,29,36,37,40]. Only one study involved fine motor movements of the hand (finger tapping) [29]. All five studies were intervention-based, with the primary objective of designing targeted tele-rehabilitation programs for the upper extremities. The effectiveness, adherence, usability, and acceptance of these designed exergame tele-rehabilitation services were evaluated in each study [28,29,36,37,40].

The duration of the interventions varied from 2 weeks to 8 weeks, with sessions occurring three times a week, and each ranging from 30 to 75 minutes per session [28,29,36,37,40]. The most frequently utilized outcome measurement tools across these five studies were the Box and Block Test (BBT) [58], Nine-Hole Peg Hole Test (9HPT) [59], the Jebsen Hand Function Test (JHFT) [60], and the MDS-UPDRS [51]. Three of the five studies reported significant improvements in gross and fine manual dexterity and grip strength, as well as the range of motion and hand movements [29,37,40]. However, two studies that utilized the 9HPT to assess gross and fine manual dexterity did not demonstrate statistically significant improvements [28,36].

Two level 4 studies [31,38] focused on lower-limb rehabilitation. Lower-limb impairments in PD are often associated with mid to late stages of the disease. One study [38] employed lateral leg movements as the primary exercise, which was delivered four times per week over five weeks, and totaled 10 hours of treatment. The other study [31] incorporated obstacle negotiation tasks on an obstacle-lined pathway, with 45-minute sessions conducted weekly over a 12-month period, using game-based elements to enhance engagement.

Across all the included studies, there were overall positive outcomes associated with GBT interventions for individuals with PD, and the interventions appeared to be feasible in the majority of cases (**see Table 4**) [33–40,42–56]. Home-based implementation was often deemed feasible and well-received [28–32,37,42,47]. Similarly, the feasibility of GBT is represented by findings on system usability, where patients and physical therapists reported that they were easy to use and well-accepted. [31,34,39]. Some noted threats to feasibility however were related to game difficulty and repetitiveness, which suggested a challenge to sustained engagement [29,34], as well as the need for individualized software enhancements [45].

**Table 4:** Clinical impact and implementation outcomes.

Author	Effectiveness, Safety, Feasibility
Albiol-Perez [26]	**Outcome measure:** Left, right and center postural control. **Effectiveness:** Within-subject improvements in postural control between baseline and final evaluation: no significant improvement in postural control, but a trend toward overall improvement, particularly in left/right positions, which are critical for falls prevention. **Safety**: NR. **Feasibility**: The system was rated highly in terms of its enjoyment, success, clarity and helpfulness.
Amprino [27]	**Outcome measure:** Statistical assessment of the immediate impacts of evaluative motor tasks. **Effectiveness:** Functional motor parameters before and after exergame training: improvement in motor function (e.g., arm swing asymmetry reduced by ~14%, movement speed and range). **Safety**: NR. **Feasibility**: The system successfully detected motor performance differences between PD patients and healthy individuals: significant differences (p < 0.05) confirmed its sensitivity to disease-specific impairments. Exergames also showed potential as engaging alternatives to traditional evaluative tasks; targeting specific motor components.
Bacha [33]	**Outcome measure:** LOS, COP, VOS. **Effectiveness:** Postural control measured pre, post-intervention and 30 days after the last session of intervention by force platform data. Significant improvements in limits of stability were observed after Kinect-based training. **Safety**: NR. **Feasibility**: The study suggests that Kinect Adventures! games training is feasible and may improve postural control (LOS) in PD, but further research with larger samples is needed to validate effects on other balance measures (COP area/speed) across different sensory conditions.
Barth [45]	**Outcome measure:** MDS-UPDRS-III, clinical measures (abduction, 5 step distance and time). **Effectiveness:** All groups showed improvements in UPDRS-III scores, with greater reductions in intervention groups vs. control group, though differences were not significant. Arm abduction angle and 5-step distance improved significantly more in intervention groups than in control (p < 0.05). Higher frequency of additional training correlated with greater improvements. **Safety**: No adverse events reported. **Feasibility**: Computer-based training, particularly with the Kinect, is feasible, safe, and well-received by patients for improving mobility, with group dynamics enhancing outcomes; however, individualized software enhancements are needed.
Brachman [35]	**Outcome measure:** Postural stability and dynamic balance. **Effectiveness:** Static and dynamic balance measured by posturography: significant improvement in dynamic balance was observed only in the exergaming group, while both groups showed improvements in static balance. **Safety**: NR. **Feasibility**: NR.
Blanc [34] (18, 3b)	**Outcome measure:** UTAUT questionnaire. **Effectiveness**: NR. **Safety**: NR. **Feasibility**: High acceptability, acceptance, and appropriateness of the system (easy to use) were reported by both physiotherapists and patients, suggesting its integration into physical therapy practice and telemedicine to be highly feasible. However, decreased intentions to use the system were reported, which was attributed to factors such as the difficulty and repetitiveness of the games, and was noted as a challenge to long-term feasibility and sustained engagement of the intervention.
Cikajlo [28] (11, 4)	**Outcome measure:** MDS-UPDRS III, JHFT, 9HPT, BBT. **Effectiveness**: Significant improvements in JHFT tasks, BBT scores (47.2 to 51.6) and MDS-UPDRS III scores (29.5 to 27.3). **Safety**: NR. **Feasibility**: High game completion and positive initial feedback suggest good feasibility for a Kinect-based upper limb tele-rehabilitation system in PD, supporting home-based use and for maintaining patient motivation.
Cikajlo [36]	**Outcome measure:** BBT, UPDRS III, writing a letter, moving light objects, JHFT, PDQ-39. **Effectiveness**: Statistically significant and clinically meaningful improvements were found without changes to medication. **Safety**: NR. **Feasibility**: NR
Cikajlo [37]	**Outcome measure:** UPDRS III, JHFT, BBT. **Effectiveness**: Evaluative tests were done before and after a 2-week exergaming intervention: significant improvements in both home and hospital groups, with some outcomes favoring the home group, supporting the program’s effectiveness. **Safety**: NR. **Feasibility**: High levels of motivation and adherence were maintained throughout the study, supporting its potential as an engaging tool for PD population. Efficiency was addressed through a comparison of home-based and outpatient exergaming program. Comparable outcomes suggested that home-based delivery methods may be a viable option.
Dauvergne [29]	**Outcome measure:** Time played, average game scores, SEQ, BAASTA. **Effectiveness**: Improvements were observed following post-music based game intervention. **Safety**: NR. **Feasibility**: The results showed the gaming program was feasible at home and reported good or excellent suitability. However, the adherence was low, which was attributed to the lack of evolution of the tele-rehabilitation tools used (bracelets and a tablet) and the absence of after-sales service.
Del Pino [49]	**Outcome measure:** EQ-5D-5L, MoCA, GDS, ADL scales, adherence rates, SUS, cost-utility. **Effectiveness:** The PD vCare group showed significantly greater improvement than the control group (p < .05), particularly in cognition (p = .016) and QoL areas: mobility (p = .008), self-care (p = .008), daily activities (p = .010), and pain/discomfort (p = .004). Adherence to vCare was high (90.5–100%) and costs were lower (€2,243.07 vs. €5,108.26). **Safety**: No adverse events reported. **Feasibility:** Demonstrated clinical validity through improved outcomes, feasibility through high patient adherence and cost-effectiveness, with significantly lower costs per patient compared to standard care.
Esculier [39]	**Outcome measure:** STST, Tinetti POMA, TUG, 10MWT, CBM, ABC, unipedal stance duration and force platform. **Effectiveness**: Significant improvement in static and dynamic balance, mobility and functional performance. **Safety**: NR. **Feasibility**: The intervention was feasible for individuals with moderate PD, showing usability and adherence. Statistically significant improvements in balance and mobility were detected, indicating its potential as an engaging tool.
Gandolfi [46] (24, 1b)	**Outcome measure:** BBS, ABC scale, 10MWT. **Effectiveness**: Significant improvements in postural stability. **Safety**: No adverse events reported. **Feasibility**: Feasibility was supported through a cost comparison, confirming the tele-rehabilitation programs as a practical alternative.
Hashemi [40] (19, 2b)	**Outcome measure:** BBS, ABC scale, 10MWT. **Effectiveness**: Improvements were observed in discriminative sensory function (HAST weight and HAST-total), wrist proprioception, gross manual dexterity and grip strength of both less and more affected hands. **Safety**: NR. **Feasibility**: Efficiency was supported through the inclusion of both supervised and non-supervised formats, suggesting exergames using Kinect appears feasible for PD tele-rehabilitation and may improve some upper limb functions.
Herz [41] (17, 3b)	**Outcome measure:** NEADL, UPDRS, 9HPT, PPT, a timed tapping task, TUG, HAMD, PDQ-39. **Effectiveness**: Significant improvements were seen in ADLs (NEADL), motor function (UPDRS), and QoL (PDQ-39), with a trend toward improved mood (HAM-D). Some improvements, particularly in UPDRS and QoL, were maintained at one-month follow-up, indicating short-term motor and non-motor benefits. **Safety**: NR. **Feasibility**: Efficiency was supported through maintained high levels of adherence over the study period, indicating the feasibility of the Wii-based home rehabilitation in PD.
Holmes [42] (17, 2b)	**Outcome measure:** COPL, ABC, number of sessions, mean active exercise time, total active exercise time. **Effectiveness**: Measured balance and balance confidence. Improvements were observed by mid-intervention but diminished post-program, suggesting potential short-term effectiveness. **Safety**: No adverse events reported. **Feasibility**: Home-based Wii balance training is feasible, well-tolerated, and maintains user interest over 12 weeks, suggesting its potential as an innovative rehabilitation strategy for PD.
Negrini [47] (20, 3b)	**Outcome measure**: Tinetti POMA, BBS, PST, FRT. **Effectiveness**: There was demonstrated effectiveness within a relatively short treatment duration (10 sessions). **Safety**: NR. **Feasibility**: This study suggests good feasibility of the proposed solution due to its low equipment cost, ease of use with minimal supervision, and positive patient opinion.
Nuic [48] (20, 1b)	**Outcome measure:** SWST duration, FOG-Q, GABS-B axial score, ABC scale, kinematic gait parameters. **Effectiveness**: There was no significant difference in primary outcomes (SWST duration) between the active and control groups after training (p = 0.61), though both groups indicated some within-group improvements. **Safety**: Nine adverse events were reported in eight participants (four in active group and four in control group). Three serious events (e.g., falls, medication-related hospitalizations) were deemed unrelated to the intervention. Five non-serious events occurred during both randomized and open-label phases. **Feasibility**: Participants maintained high levels of motivation and adherence to the program consistently throughout the duration of the study.
Nuic [30] (14, 4)	**Outcome measure:** Perceived difficulty, competence, interest, fatigue, affects, acceptability, FOG-Q, ABC, GABS-B axial score, UPDRS, Parkinson motor disability UPDRS III, ADL, UPDRS II. **Effectiveness**: Significant improvements were observed in post training, supporting the use and effectiveness of customized video game interventions. **Safety**: No adverse events reported. **Feasibility**: Efficiency was observed through high feasibility, acceptability and satisfaction rates.
Palacios-N. [31] (11, 4)	**Outcome measure:** 10MWT. **Effectiveness**: Significant improvements following the intervention. **Safety**: No adverse events reported. **Feasibility**: The rehabilitation game was well-accepted, easy to use, and described as motivating by participants. The collected feedback from participants lends support for its feasibility for home-based use.
Pelosin [32] (11, 4)	**Outcome measure:** Posturographic parameters (sway area and path, x- and y-axis oscillations), BBS. **Effectiveness**: Measured postural and balance before and after a 5-day exergame-based training program, demonstrating a significant decrease in all posturographic parameters in both PD and control groups. This result shows improved postural stability in PD patients to a level comparable with baseline in non-symptomatic individuals. BBS scores also improved after training in PD patients. **Safety**: NR. **Feasibility**: The study indicates good feasibility due to its short effective training period, low cost, engaging nature, and potential for home use with augmented feedback.
Cornejo-T. [38]	**Outcome measure:** Gait speed, ABC Scale, MDS-UPDRS. **Effectiveness**: Functional improvements in individuals with PD. **Safety**: No adverse events reported. **Validation/Feasibility**: Participants maintained high levels of motivation and adhered to the program consistently throughout the duration of the study. Real-time communication and remote setting adjustments supported an ongoing engagement.
Vallabhajosula [43]	**Outcome measure:** FES-I, MiniBEST test, 2MWT, gait parameters (COP, step length/time/velocity, etc.). **Effectiveness:** Measured changes in postural control, gait, endurance, and clinical balance outcomes. Significant improvements were observed in FES-I, MiniBEST test, 2MWT, gait parameters. However, only a few of these improvements were maintained during the post-intervention phase. **Safety**: NR. **Feasibility**: This case study suggests the multimodal training protocol was feasible to implement. However, the results only indicated potential benefits for static and dynamic postural control but not for gait, cognition, endurance and clinical measures of balance. These findings are limited due to the single-participant design.
Zeigelboim [44]	**Outcome measure:** VADL, ABC Scale. **Effectiveness**: Measured balance, independence, and confidence in daily activities using VADL and ABC scale, which indicated significant improvements. **Safety**: No adverse events reported. **Feasibility**: NR.

2MWT: 2 Minute Walk Test, 9HPT: Nine-Hole Peg Test, 10MWT: 10-Meter Walking Test, ABC: Activities-specific Balance & Confidence Scale, ADL: Activities of Daily Living, BBS: Berg Balance Scale, BBT: Box-Block Test, CBM: Community Balance & Mobility Assessment, COP: Sway of Center of Body-pressure, EQ-5D-5L: EuroQoL-5D-5L, FES-I: Falls Efficiency Scale-I, FOG-Q: Freezing of Gait Questionnaire, FRT: Fall Risk Testing, GABS-B: Gait & Balance Scale Part B, HAST: Hand Active Sensation Test, HAMD: Hamilton Depression Rating Scale, LOS: Limits of Stability, MDS: Movement Disorder Society, MiniBEST: Mini Balance Evaluation System Test, NR: Not Reported, PDQ: Parkinson’s Disease Questionnaire, POMA: Performance-Oriented Mobility Assessment, PST: Postural Stability Testing, QoL: Quality of Life, SEQ: Suitability Evaluation Questionnaire, STST: Sit-to-stand Test, TUG: Timed Up & Go, UPDRS: Unified Parkinson’s Disease Rating Scale, UTAUT: Unified Theory of Acceptance & Use of Technology, VADL: Vestibular Disorders Activities of Daily Living, WPST: Wrist Position Sense Test, VOS: Vertical Optokinetic Stimulus.

While the findings from the studies suggest that the interventions were well-accepted and feasible, the consistency and magnitude of the therapeutic effects of GBT varied across studies. For instance, some interventions demonstrated either only a trend towards improvement [26], short-lived effects [49], or non-significant differences compared to control groups for primary outcomes [45,52,55]. Additionally, four studies provided insights into the effectiveness of GBT beyond the active intervention period. One study [41] showed some longer-term effectiveness, whereby improvements in motor function and quality of life were maintained at a one-month follow-up. Overall, positive impacts were observed but the therapeutic gains over time varied in those studies where documented.

### Safety and feasibility

Of the 24 studies, 8 studies of varied methodological quality indicated that the GBT interventions were safe, with no adverse events reported [30,31,38,42,44–46,49]. Only one study [48] reported on nine events in eight of their participants (four in the active group and four in the control group). However, these events were deemed unrelated to the intervention, including three serious events (e.g., falls, medication-related hospitalizations) and five non-serious events. The remaining included studies did not report on safety.

There are several factors reported across the included studies that contribute to the feasibility of GBT to support physical therapy in home settings, such as end-user experience, adherence, perceived use, and cost-effectiveness. For instance, participants across studies rated the systems to be highly enjoyable [26], and exhibited high levels of motivation and adherence [27,28,37,38,41–43,48], which included high rates of game completion [28]. Relatedly, home-based implementation was often deemed feasible and well-received [28–32,37,42,47], and that systems were easy-to-use and well-accepted by both patients and physical therapists [31,34,39]. Importantly, participants expressed satisfaction with the interventions [30,31,39]. As noted above, studies also provided evidence on their cost-effectiveness compared to in-person approaches [32,46,47,49].

Among the seven studies that reported on the adherence levels of individuals with PD to GBT programs, four studies [29,34,37] utilized customized gamification modules without specifying a product name except for one [49], while one study [42] used the Nintendo Wii. Two studies [38,48] used the Microsoft Xbox 360 Kinect gaming console. Additionally, among the seven studies, two reported low adherence levels and decreased intentions to use the tele-rehabilitation program. The lack of adherence was attributed to factors such as the difficulty and repetitiveness of the games [34], as well as the lack of evolution of the used tele-rehabilitation tool (comprising bracelets equipped with motion sensors and a tablet) and the absence of after-sales service [29]. The other five studies reported that their participants maintained high levels of motivation and adhered to the exergaming programs consistently throughout the duration of the studies [37,38,42,48,49].

### Hardware and software modules

A comprehensive list of the software modules and hardware devices utilized in the included studies are listed in Tables 5 and 6, respectively. Eleven of the 24 included studies [1 level 1b, 2 level 2b, 4 level 3b, and 4 level 4] used commercially available games [26,29,32,33,36,39,41–43,46,47], with most being those produced by the Nintendo Co., Ltd. (www.nintendo.com), which was the Nintendo Wii game console accompanied by the Wii Balance Board (7 out of 11) [26,32,39,41,42,46,47]. This was followed by systems developed by Microsoft’s Xbox 360 game console (www.xbox.com; 3 out of 11) paired with the Kinect camera [33,36,43].

**Table 5:** Software features.

Author	Type: Customized or commercial game Game company: if commercial. Game names	Additional Functional Features
Albiol-Perez [26]	**Type**: Commercial. **Product name**: Wii. **Games**: Not reported.	**Game tool:** VR. **Game challenge customization for patients**: Yes. **Exercise library**: Yes. **Patient/PT communication via software**: No. **Additional functions**: (1) Enables patients to customize configuration parameters, (2) provides the exercise overview at the end of each session, (3) grants PT access to patients’ exercise overview.
Amprino [27]	**Type**: Customized Games Using Commercial Platform. **Product name**: Microsoft Azure Kinect. **Games**: Not reported.	**Game tool:** VR. **Game challenge customization for patients**: Yes. **Exercise library**: No. **Patient/PT communication via software**: No. **Additional functions**: Configuration file available for each exergame to set specific game parameters, making it easy to adjust exercise difficulty (Easy, Medium, Hard) to suit a patient’s motor condition and rehabilitation goals.
Bacha [33]	**Type**: Commercial. **Product name**: XBOX Kinect. **Games**: Kinect Adventures (20,000 leaks, Space Pop, Reflex Ridge, River Rush)	**Game tool**: VR. **Game challenge customization for patients**: No. **Exercise library**: No. **Patient/PT communication via software**: No. **Additional functions**: (1) Five chances in each game, (2) PT present at first to provide guidance and familiarize participant, (3) the games stimulated the individuals to move in different directions in a fast and controlled way, walk in different directions, move their centre of mass, sit down and jump, move their upper and lower body in a coordinated way and move their upper body in the three planes of movements.
Barth [45]	**Type**: Customized module Using Commercial Platform. **Product name**: Microsoft Kinect. **Games**: Coconuts, Stars, Balls, Balloons	**Game tool:** VR. **Game challenge customization for patients**: No. **Exercise library**: No. **Patient/PT communication via software**: No. **Additional functions**: (1) Special virtual reality training game utilizing the Microsoft Kinect® camera was developed in collaboration with an experienced software company tailored for patients with PD, (2) software includes guiding instructions for the whole game, as well as previews for all the movements the patients have to perform within the different games making it completely self-explanatory.
Brachman [35]	**Type**: Customized. **Product name**: NA **Games:** Boat, Colours, Froglet, Football player, Burro, Bicycles, Fruits	**Game tool:** VR **Game challenge customization for patients**: Yes. **Exercise library**: Not specified. **Patient/PT communication via software**: No. **Additional functions**: Involves physiotherapist supervision during training sessions to ensure safety.
Blanc [34]	**Type**: Customized. **Product name**: NA. **Games:** Serious games.	**Game tool:** VR. **Game challenge customization for patients**: Automatic, increase due to several parameters (duration of session, frequency of exercise, etc.) **Exercise library**: Not specified. **Patient/PT communication via software**: Yes, format not specified. **Additional functions**: (1) Allows performing repetitive movements with large amplitude and defined pace, (2) provides visual feedback through a visual representation of a vessel catching coins and stars. Offers auditory feedback based on the performed movements, (3) facilitates connection with field professionals through a secure telemedicine platform, (4) enables remote monitoring.
Cikajlo [36]	**Type**: Customized. **Product name**: NA. **Games:** Fruit Picking.	**Game tool:** VR. **Game challenge customization for patients**: Yes, automatic. **Exercise library**: Not specified. **Patient/PT communication via software**: No. **Additional functions**: (1) Records and stores data on a local computer during user/patient exercise sessions, (2) allows medical professionals to remotely review data for planning and following exergame-based therapy, (3) enables system administrator to update gamification platforms, add new medical professionals, change access privileges, and grant physician access, (4) allows saved data to be exported to other software tools for further analysis.
Cikajlo [37]	**Type**: Customized. **Product name**: NA. **Games**: Pick and Place game.	**Game tool:** VR. **Game challenge customization for patients:** No. **Exercise library**: No. **Patient/PT communication via Software**: No. **Additional functions**: (1) Utilizes a client-server model, with a server and database hosting the front-end interface for remote therapists and a client running the exergame, (2) synchronizes data after each exercise session to maintain data integrity and consistency.
Cikajlo [28]	**Type**: Customized module Using Commercial Platform. **Product name**: Microsoft Kinect. **Games**: Fruit Picking	**Game tool:** VR. **Game challenge customization for patients**: Yes, automatic. **Exercise library**: No. **Patient/PT communication via software**: No. **Additional functions**: (1) “Fruit picking” was designed as a target based task, requesting from the user to raise the arm and pick the moving targets, apples growing slow/fast on the tree. More successful users advanced faster to the higher levels.
Dauvergne [29]	**Type**: Customized. **Product name**: NA. **Games:** Music-based serious game.	**Game tool**: VR. **Game challenge customization for patients**: Automatic. **Exercise library**: No. **Patient/PT communication via software**: No. **Additional functions**: (1) Incorporates different levels of difficulty within the game, with scores between 5 and 100 points calculated at the end of each level, (2) unlocks subsequent levels based on the score achieved, which is converted into a number of stars displayed on the screen accompanied by motivating messages, (3) allows automatic unlocking of the next level after 5 successful trials at the same level.
Del Pino [49]	**Type:** Customized. **Product Name:** vCare system. **Games:** Custom serious games developed as part of the vCare system.	**Game tool:** VR. **Game Tool:** Virtual Exercise Coaching. **Game challenge customization for patients:** Yes. **Exercise library:** No. **Patient/PT communication via software:** No. **Additional functions:** (1) Virtual coach avatar provides motivational prompts and interaction, (2) providers remotely monitor progress and adjust/customize weekly exercise programs, (3) vCare system includes a structured adherence tracking dashboard.
Esculier [39]	**Type**: Commercial. **Product name**: Wii Fit. **Games:** Table Tilt, Ski Slalom, Balance Bubble, Ski Jump, Penguin Slide.	**Game tool:** VR. **Game challenge customization for patients**: Automatic. **Exercise library**: No. **Patient/PT communication via software**: No. **Additional functions**: (1) Participants received a logbook. Training progression within the activities was carried out by the Wii Fit game itself. Increased difficulty levels could be permanently unlocked secondary to good performances, as well as temporary levels according to daily results, (2) a motivational telephone call was made by one of the researchers every week and to ensure that the subjects were doing well. Participants were encouraged to contact the research team to ask any questions about the programme or to request assistance with technical problems.
Gandolfi [46]	**Type**: Commercial. **Product name**: Wii. **Games:** Wii Fit.	**Game tool:** VR. **Game challenge customization for patients**: Manually by PT through game selections. Automatically by game. **Exercise library**: No. **Patient/PT communication via software**: No. **Additional functions**: (1) Supports video calls during intervention sessions, (2) provides full tracking of patients’ performance, ensuring correct implementation of exercises, (3) offering real-time visual feedback to patients on their performance.
Hashemi [40]	**Type**: Commercial. **Product name**: Kinect. **Games:** Not specified.	**Game tool:** VR. **Game challenge customization for patients**: Yes. **Exercise library**: No. **Patient/PT communication via software**: No. **Additional functions**: (1) Allows for games with customized and random challenges, (2) offers visual cues about patients’ performance, (3) provides audio feedback to inform and encourage patients during exercises, (4) initiates automatic running of exercise items sequentially when the patient turns on the system, (5) structures each session with a warm-up, main exercise item, and evaluation tests, (6) allows patients to pause, rest, and resume if feeling tired during exercising, (7) generates reports on patient performance through software, (8) conducts evaluation tests to determine the difficulty level of exercises for subsequent sessions.
Herz [41]	**Type**: Commercial **Product name**: Wii. **Games:** Tennis, Bowling, Boxing	**Game tool:** VR. **Game challenge customization for patients**: Not specified. **Exercise library**: No. **Patient/PT communication via software**: No. **Additional functions**: Not specified.
Holmes [42]	**Type**: Commercial **Product name**: Wii. **Games:** WiiFit plus, Balance bubble, Table Tilt, Soccer Heading, Tightrope Tension, Penguin Slide, Ski Slalom, Snowboard Slalom.	**Game tool:** VR. **Game challenge customization for patients**: Yes. **Exercise library**: No. **Patient/PT communication via software**: No. **Additional functions**: (1) Enables full tracking of patient performance, including monitoring the correct implementation of exercises, (2) provides real-time visual feedback to patients regarding their performance.
Negrini [47]	**Type**: Commercial. **Product name**: Wii. **Games:** Wii Fit Balance Board: Table Tilt, Ski Slalom, Balance Bubble, Ski Jump and Penguin Slide.	**Game tool:** VR. **Game challenge customization for patients**: Not specified. **Exercise library**: No. **Patient/PT communication via software**: No. **Additional functions**: Not specified.
Nuic [30]	**Type**: Customized. **Product name**: NA. **Games:** Toap Run.	**Game tool:** VR and Virtual Exercise Coaching. **Game challenge customization for patients**: Manually, by PT. **Exercise library**: No. **Patient/PT communication via software**: No **Additional Functions:** (1) Utilizes visual cueing through avatar movements along the trajectory to guide patients during exercises, (2) incorporates auditory cueing with rhythmic music synchronized to the velocity of the avatar displacement.
Nuic [48]	**Type**: Customized module using Commercial Platform. **Product name**: Microsoft RGB-D Kinect Motion Sensor. **Games:** Toap Run.	**Game tool:** VR. **Game challenge customization for patients**: Yes. **Exercise library**: No. **Patient/PT communication via software**: No. **Additional functions**: (1) The session duration, number of movements, and success rate were automatically recorded, allowing the investigator to follow and individually tailor the game difficulty (easy, medium, hard), (2) designed to treat gait and balance disorders for PD patients, (3) to play, the patient had to perform large amplitude and fast movements of all four limbs, pelvis and trunk, in response to visual and auditory cueing, to displace an avatar to collect coins and avoid obstacles to gain points.
Palacios-Navarro [31]	**Type**: Customized. **Product name**: NA. **Games:** Not specified.	**Game tool:** VR. **Game challenge customization for patients**: Yes, manually by PT. **Exercise library**: Not specified. **Patient/PT communication via software**: No. **Additional functions**: (1) Allows patients to set configuration parameters, (2) provides exercise overview at the end of each session. Grants PT access to patients’ exercise overview.
Pelosin [32]	**Type**: Commercial. **Product name**: Nintendo Wii-Fit. **Games:** Wii-Fit.	**Game tool:** VR. **Game challenge customization for patients**: Automatic. **Exercise library**: No. **Patient/PT communication via software**: No. **Additional functions**: The complexity of the exercises was progressively increased and the maximum game-level reached depended on the subjects’ ability.
Cornejo Thumm [38]	**Type**: Customized module using Commercial Platform. **Product name**: Microsoft Kinect. **Games:** Not specified.	**Game tool:** VR. **Game challenge customization for patients**: Yes, manually by PT. **Exercise library**: Not specified. **Patient/PT communication via software**: Yes. **Additional functions**: (1) Remote monitoring software (Google Chrome remote desktop tool) and Skype were also installed to enable visual and auditory communication during training, (2) allows trainer to monitor participant’s movement, provide feedback in real-time, and manage parameters remotely. Training settings were controlled remotely to change the level of challenge.
Vallabhajosula [43]	**Type**: Commercial. **Product name**: XBOX Kinect. **Games:** Kinect Sports Season 1 DVD: Bowling, Boxing, Table Tennis.	**Game tool:** VR. **Game challenge customization for patients**: Yes. **Exercise library**: No. **Patient/PT communication via software**: No. **Additional functions**: (1) Based on the participant’s performance and choice, all the Kinect games were played at the easiest level throughout the intervention, (2) participants could choose from multiple game designs targeting stepping, trunk rotation, and balance.
Zeigelboim [44]	**Type**: Commercial. **Product name**: Wii. **Games:** Soccer Heading, Table Tilt, Tightrope Walk, Ski Slalom.	**Game tool**: VR. **Game challenge customization for patients**: No. **Exercise library**: No. **Patient/PT communication via software**: No. **Additional functions**: (1) Utilizes the Wii balance board to engage reactive and proactive strategies of postural control, (2) offers visual feedback to enhance performance and motivation in completing the games.

*GM: Gamification module, PT: Physical Therapist, VR: Virtual Reality.

**Table 6:** Hardware features.

Author (Year)	Customized Hardware (yes/no)	Sensor Type	Supporting Platform	Additional Hardware
Albiol-Perez [26]	No	Nintendo Wii Balance Board	Tablet	No
Amprino [27]	Yes	Microsoft Azure Kinect Camera	Computer	Monitor
Bacha [33]	No	Xbox Kinect	Xbox 360	Virtual reality glasses
Barth [45]	No	Kinect Camera	Computer	LCD Screen
Brachman [35]	Yes	Kinect Camera and a Custom-made Force Platform	Computer	65-inch screen 2 meters away from participant
Blanc [34]	Yes	Bracelets equipped with inertial motion sensors	Tablet	Velcro strips
Cikajlo [28]	No	3D Infrared Camera (Kinect Sensor)	Computer	Unity 3D game engine
Cikajlo [36]	No	Kinect Camera	Computer	LCD screen
Cikajlo [37]	No	Leap Motion Controller	Computer	Cloud server, Camera connects to high speed USB of computer and requires graphic adapter (e.g., Nvidia GeForce series). Infrared camera requires light calibration or constant light conditions to operate properly.
Cornejo Thumm [38]	Yes	VR Stimulation	Computer	TV screen, treadmill.
Dauvergne [29]	No	Touch Screen	Tablet	No
Del Pino [49]	Yes	N/A	Tablet	TV screen
Esculier [39]	No	Nintendo Wii Balance Board	Nintendo Wii Console	Monitor, TV.
Gandolfi [46]	No	Nintendo Wii Balance Board	Nintendo Wii Console	No
Hashemi [40]	No	Kinect Camera	Computer	Monitor, computer 2 or 3 meters away from rehabilitation station.
Herz [41]	No	N/A	Computer	No
Holmes [42]	No	Nintendo Wii Balance Board	Nintendo Wii Console	Screen
Negrini [47]	No	Nintendo Wii Balance Board	Nintendo Wii Console	Monitor
Nuic [48]	Yes	RGB-D Kinect Motion Sensor (version 2, Microsoft)	Laptop	HD TV screen
Nuic [30]	No	Kinect Camera and Force Platform	Computer	Laptop, TV 2 meters away from patients.
Palacios-Navarro [31]	No	Kinect Camera	Computer	Computer, Cloud, monitor, Microsoft Kinect Device, 2 GB Ram, graphic card compatible with Direct X9 and processor Intel Pentium 4 3.0 Ghz, Screen 2 meters away from patients.
Pelosin [32]	No	Nintendo Wii Balance Board	Wii Console	Monitor, TV.
Vallabhajosula [43]	No	Xbox Kinect	Xbox 360	Monitor, TV.
Zeigelboim [44]	No	Nintendo Wii Balance Board	Wii Console	Screen

The Nintendo Wii is a gaming console renowned for its innovative motion control system, which is showcased by the Wii Remote, Wii Nunchuk, and Wii Balance Board. The Wii Balance Board is an accessory that is designed to be used with the Wii console, enabling players to participate in games using their balance and physical movements. The Wii Balance Board enhances interactivity and immersion by accurately detecting and responding to players’ movements. The Xbox 360, developed by Microsoft, is a gaming console known for its extensive gaming library and immersive experiences. One notable addition to the Xbox 360 is the Kinect camera, which is a motion-sensing device. The Kinect camera, with its advanced depth sensing, enables controller-free interaction, by tracking players’ body movements, and translates them into game actions.

Among the 13 studies [2 level 1b, 2 level 2b, 3 level 3b, and 6 level 4] with custom-made games, 9 studies [27,28,30,31,35,36,38,45,48] developed their games utilizing the Kinect Camera sensory module. The Kinect camera offers depth sensing, skeletal tracking, and motion capture capabilities for game development. Microsoft provides software development kits (SDKs) and tools that grant access to the Kinect’s sensor data. Developers can incorporate gesture recognition, body tracking, and interactive features into their custom games, enabling the creation of unique and personalized gaming experiences. Additionally, one study developed its own bracelet-type sensory module [34] using an inertial motion sensor, one study [29] utilized the sensory inputs of a touch screen, one [49] used a tablet-based virtual coaching system called vCare that delivered customized games without the use of motion sensors or depth cameras, and one [37] relied on the sensory data of a Leap motion controller. The Leap Motion controller, introduced by Leap Motion Inc. (www.ultraleap.com/), is a compact device known for its precise hand and finger tracking. It enables intuitive gesture-based interaction with computers and digital devices, offering fine-grained control and immersive experiences. The commonly included hardware platforms used to run the software applications to process the data and to display it on a monitor were computers, tablets, and Nintendo Wii consoles.

Game-based solutions adapted in the rehabilitation interventions in all 24 studies were non-immersive VR-based serious exergames, except in one level 4 study [30], and one level 2b study [49] that used a combination of both non-immersive VR and virtual exercise coaching with the gamification techniques. Eleven of the designed gaming interventions included offline or real-time interactive communication and connection, as well as streaming software applications (i.e., Skype, Zoom), which allowed for the therapist/ medical professional to provide ongoing supervision and/or real-time visual feedback to patients on their performance, full tracking of patients’ performance while completing the session, and monitoring if the correct implementation of exercises was being performed [26,27,32,34,35,38,40,43,46–48]. In contrast, the other 12 studies [1 level 1b, 1 level 2b, 4 level 3b, and 6 level 4] consisted of tele-rehabilitation systems designed as a client-server model, where the users/patients self-run the session at their convenience, and the system records and stores the data; enabling the therapist/ medical professional to review data remotely and plan/ follow exergames-based therapy [28–31,33,36,37,39,41,42,44,45].

### Applied gamification elements

Several game designs used combinations of different elements and techniques. For instance, some exergame designs introduced persons with PD as avatar characters within the game and assigned them target-based tasks (i.e., fruit picking, finger tapping task, and moving small virtual objects) with the simultaneous addition of auditory and/or visual feedback. For instance, the level 4 study by Nuic and colleagues [30] designed a video game consisting of a scenario of the patient moving to induce displacement of a small animal (avatar) in real-time environments to avoid obstacles and collect points related to visual and auditory feedback. Ten out of 24 studies [1 level 2b, 3 level 3b, 6 level 4] focused on the implementation of a single game [26,28–31,34,36–38,44], while the other 14 studies [3 level 1b, 4 level 2b, 4 level 3b, 3 level 4] provided multiple different games aiming to increase motivation and acceptance of users by inhibiting boredom [27,32,33,35,39–43,45–49].

### Game dynamics

One study [49] did not provide a clear description of the implemented game dynamics by participants when undergoing GBT other than to state the activities were personalized. For the remaining 23 studies, all implemented tasks were based on six main categories of game dynamics (activities) for therapeutic games: (1) free movements (5/23 or 22%) [38,42–44,46]; (2) reach and hit the target (8/23 or 35%) [30–33,35,40,41,43]; (3) catch and pick up the target (8/23 or 35%) [33–39,48]; (4) carry and move the target (12/23 or 52%) [26,28,33,35,36,39,41–44,46,48]; (5) follow and track the moving target (3/12 or 25%) [35,40,44]; and (6) shift body weight right or left (5/23 or 22%) [27,35,39,42,46]. All six categories of game dynamics were used to design the therapeutic exergames that focused on whole-body rehabilitation. Activities such as reaching and hitting the target, catching and picking up the target, carrying and moving the target, and following and tracking the target were used in the design of upper-limb rehabilitation-based exergames. In one study [31] that specifically targeted lower-limb rehabilitation, the therapeutic exergames were designed around a single game activity involving reaching and hitting the target.

## Discussion

The objective of this scoping review was to comprehensively examine the available literature on the GBT systems developed to facilitate physical therapy for persons with PD in home settings. This review aimed to explore the design and technological components of these systems to extract implementation considerations, and to describe their impact on physical function and patient well-being. The 24 articles included in this scoping review were comprised mostly of low levels of evidence with poor to fair methodological quality, which indicates more rigorous study designs are required to elevate the evidence for use of these technologies. With regard to study design, all were quantitative studies that aimed to examine the extent to which GBT approaches yielded comparable outcomes to traditional standard rehabilitation services. In the majority of the included studies, sample size sufficiency was often viewed as inadequate or being relatively small but was acknowledged in the context of each study’s limitations section. As a result, the low methodological quality paired with small samples limits the quality of evidence for the PD population.

When examining the characteristics of the included participants across studies, the majority were in the earlier or mid-stages of the disease progression, and all were cognitively intact. This sampling approach may have been undertaken for safety reasons (e.g., later stages at higher risks for adverse events or physically unable to participate), and because those in the earlier or mid-stages still have the capability to retain better control over their movements, and physical therapy is likely more effective in improving or maintaining functional abilities. It has been noted that while rehabilitation is important to provide throughout the disease’s progression, initiating it early contributes to a foundation that makes interventions in later stages more effective. [61] Although the included participants across studies did not have any significant cognitive impairments, it is known that cognition does become increasingly impaired as the disease progresses, which includes multi-tasking, word recall, problem-solving and maintaining focus, which are all relevant functions to participate in GBT. As such, undertaking re-assessments of cognition in patients over time is warranted to ensure they are able to continue to participate in GBT in the long-term, and to perhaps adjust gaming complexity to ensure it does not overwhelm participants, and lead to adverse effects.

Relatedly, the lack of studies on later stages suggests there may be opportunities to explore the application of GBT physical therapy for these patients, as they may face additional barriers to accessing traditional forms of physical therapy since lowered physical function, among other factors, might make it more difficult for patients to travel to clinics to receive specialized care. [62] Our findings indicate this is a knowledge gap that warrants further investigation to enhance outcomes of those in the later stages. As well, more longitudinal studies may elucidate how GBT influences PD progression over time.

Based on the included studies, it appears participants with PD are able to tolerate interventions ranging from 1 to 8 weeks, with some studies having people participate up to three times a week for a minimum of 30 minutes and a maximum of 75 minutes per session. This duration of physical activity aligns with existing guidelines for the PD population [63], which recommends that this population engage in aerobic exercise three days a week for at least 30 minutes per session of continuous or intermittent movement at a moderate or vigorous intensity. As well, persons with PD should undergo strength training 2–3 non-consecutive days a week. Other components involve activities related to balance, multi-tasking, agility and stretching. Based on our review, it appears that a physical therapy-prescribed GBT approach, properly calibrated in terms of duration, intensity, and game design can serve to help persons with PD engage in clinically impactful outcomes while also serving to put individuals on a path to regular physical activity since some of the commercially available options can be continued post-physical therapy intervention.

Additionally, the findings regarding safety are encouraging, with the majority of studies reporting no adverse events associated with GBT interventions. Only one study reported any adverse events, which was considered unrelated to the intervention. Regardless, and as noted above, it may be prudent to periodically re-assess physical and cognitive abilities to ensure GBT remains a safe modality as the disease progresses. While other included studies did not comment on adverse events, which implies none occurred, it is still important that researchers explicitly document the absence of these to help raise confidence in GBT as well as increase enthusiasm for it by minimizing potential safety concerns, which need to be especially accounted for if exercises are being done at home without a therapist being there to oversee the patient. Overall, physical therapists interested in prescribing GBT can assume that the associated risks for its’ use are low, and likely can be utilized by patients following a standard physical and cognitive assessment, along with providing appropriate training and guidance.

Critically, the high rates of adherence noted across studies highlights the motivating and appealing features of GBT for persons with PD. However, a number of articles in this review highlighted the importance of the need to make appropriate exercise and difficulty adjustments to the exercise program throughout the intervention period to sustain users’ interest and adherence to the home-based program. In addition, the use of auditory and visual feedback (cues) in the game structure may also have beneficial impacts for providing relevant feedback to patients; thereby keeping them engaged. Interestingly, this does not necessitate the need for the use of a wide variety of games to sustain interest since 10 of the 24 includes studies focused on the implementation of a single game or limited set of games [26,28–31,34,36–38,44]. For instance, Zeigelboim et al. [44] used four games provided by the Nintendo Wii that provided visual feedback on performance (soccer heading, table tile, tightrope walk, and ski slalom), where significant improvements in gait and balance were noted in the tightrope walking and ski slalom games over the course of 20 sessions. Overall, our analysis of the application systems in terms of the game design elements and techniques applied, it is apparent they are often easy to implement and physical therapists can use game components and techniques in a number of ways to help their clients. The findings of this review further suggest that within the development of GBT, more complex game components and a combination of multiple different games are rarely used. Hence, providers should feel confident that using single, easy-to-use and broader-concept games will likely make it easier for patients to initially follow at home.

With regard to hardware and software modules employed, most of the included studies customized their own games while the rest utilized those from commercially available games. The two most popular platforms were the Nintendo Wii paired with the Wii balance board, as well as Microsoft Xbox 360 paired with the Kinect camera. Moreover, the systems that incorporated customized games primarily utilized the Xbox Kinect Sensor. As such, commercial systems appear to be easily used within one’s home, and are adaptable to enable customized games designed to provide individuals with PD with therapeutic benefits. A challenge with the commercially available systems, however, is that both Microsoft Xbox and the Nintendo Wii were discontinued, which will make access to these systems to both patients and providers more challenging as time continues. Regardless, commercial systems are those that are relatively low-cost to purchase as noted by several of the authors across the included studies, and lend themselves for easier access by patients and clinicians. When systems are customized, this adds a layer of complexity and cost, with one study [38] pairing a treadmill with the GBT, and offered patients an optional harness attached to the ceiling for safety, and the estimated cost of the system with the treadmill was $2,000 (currency not specified).

Conversely, a key advantage of developing customized gaming modules, even when using the sensory components of commercial gaming systems, is their independence from the game market; allowing for tailored rehabilitation experiences. For new research groups, selecting the right motion-sensing technology requires balancing innovation with practicality. Established platforms like the Wii and Kinect used in the included studies demonstrated efficacy, albeit in studies of low methodological quality, but also have the advantage of clinical familiarity. The challenge moving forward is to determine approaches for incorporating newer technologies such as including VR-based systems, artificial intelligence (AI)-powered motion capture, and wearable sensors, which offer greater accuracy and adaptability. A hybrid approach that maintains the accessibility of Wii/Kinect while integrating emerging technologies may be the most effective strategy for advancing GBT.

Our analysis of current GBT systems shows a preference for easy-to-implement components like target-based tasks, avatar navigation, and simple motion tracking with feedback. While these enhance usability for people with PD, they often limit adaptability and complexity, which are crucial as PD symptoms progress. Many systems focus on single-limb or single-task exercises, potentially reducing long-term engagement and functional improvement. Future research should prioritize modular, multi-activity games that adapt to evolving motor impairments and increase in complexity. Structured progression—from simple to full-body movements—could support sustained engagement and holistic therapy. Seamless task transitions and personalized challenges can also serve to optimize motor learning and maintain motivation.

While not explored in the included studies, further work exploring community-based gamification—such as multiplayer or remote cooperative games—may hold promise for improving motivation and adherence in prescribed physical therapy for persons with PD given the reduced levels of community participation that occurs as the disease progresses [64]. Exploring how social connectivity influences outcomes can inform both physical and psychological rehabilitation strategies. To further enhance effectiveness, systems should integrate adaptive difficulty, machine learning-driven personalization, and multi-modal feedback (e.g., haptic, proprioceptive). Several of the included studies in the present review emphasized the need for dynamic exercise adjustments to sustain user interest and adherence. Eight studies featured cueing systems—mostly using commercial modules like balance boards—to aid gait and balance. Cueing types included verbal encouragement and real-time feedback, which are all effective in improving motor performance. As symptoms worsen, cueing strategies should evolve from simple rhythmic prompts to multimodal support, addressing both motor and cognitive decline. Including cognitive-motor tasks can further improve engagement and outcomes in PD rehabilitation.

From our review, we have mapped out a range of GBT options that range from being relatively simple, and those that are much more complex in their implementation. However, it is difficult to extract ideal implementation considerations given the variety of GBT approaches used with the PD population in terms of the ideal set-up. The lack of qualitative findings related to end-user experiences, including the perspectives of providers, is a glaring gap in knowledge on ways on what patients and providers might view as the ideal set-up to meet their respective needs, and what would incentivize them to adopt and continue to use GBT.

A systematic review on GBT for neurological disorders [65], which included one PD study identified in this review [40], reported on a number of challenges (i.e., medical constraints, technical issues, safety of the technology, individual and social attitudes) and opportunities (i.e., medical opportunities, individual and societal attitudes towards new technologies, financial opportunities) associated with GBT. While many of the noted issues have applicability to the PD population, there are additional nuances to the PD population that need to be better contextualized since it is progressive in nature with unique physical and cognitive features compared to other neurological conditions (i.e., stroke).

Critically, pairing a qualitative approach with an underlying theory may also be useful for helping to pinpoint these implementation considerations. For the present review, there appeared to be little attention paid to applying models and theories in establishing GBT interventions to demonstrate or prove the effects of game design elements on users’ behaviors or therapeutic outcomes. Only one study used an integrated UTAUT model to evaluate the usability of a digital self-rehabilitation device in persons with PD [34]. Doing so may help provide additional insights on why some of the participants in some studies had low interest/ adherence [29,34], while others had high levels [37,38,42,48], as well as elucidate what is required to optimize the delivery of GBT for the PD population.

In summary, the findings of this review revealed that almost all of the evaluated GBT interventions highlighted their technical feasibility, efficacy, and acceptance in home settings. This aligns with another review noting that tele-rehabilitation is a feasible approach for the PD population [66] and with the conceptual findings of a recent meta-analysis conducted on the stroke population by Aminov et al. [67], which found that both types of exergames, customized and commercially available off-the-shelf systems, are effective in enhancing upper extremity motor function, activity, and social participation when compared with conventional therapy. The evidence from our review and others [66,67] supporting its’ use can be used to build increased acceptance by a variety of stakeholders (e.g., people with PD, clinicians, decision-makers, etc.); thereby facilitating its’ implementation [68]. While the results of this scoping review also provides an initial roadmap of the types of patients, GBT technologies, and parameters of clinical efficacy for providers to refer to as a means of starting their own GBT intervention for their patients, there is clearly a need for additional research to better clarify the ideal implementation considerations of GBT for PD.

### Limitations

Several limitations should be acknowledged in this scoping review. Firstly, the inclusion criteria were limited to English-written articles, potentially leading to the omission of relevant studies in other languages. Secondly, our search strategy may not have captured all relevant literature, potentially affecting our interpretations of the results. It is important to note that the primary purpose of this scoping review was not to assess the quality of evidence per se, but rather to gain insights into the current approaches used in the PD population and extract key implementation considerations for home-based settings. As such, the findings of this review can serve as a valuable roadmap for the design of future research studies in PD and offer broader implementation considerations to support the adoption and integration of GBT to enhance physical therapy outcomes.

## Conclusion

This scoping review provides an overview of the current literature concerning the utilization of GBT systems developed to facilitate physical therapy exercises among individuals with PD. GBT is increasingly being employed to enhance different functional skills such as physical activity, mobility, and balance, among individuals with PD in various practice settings including rehabilitation centers, hospitals, community health centers, and homes. Based on the findings from this review, the combination of exergames with tele-rehabilitation presents an intriguing alternative to conventional therapy that would benefit from more research given its potential to enable ongoing monitoring by healthcare providers and to increase the motivation of individuals with PD to actively participate in their rehabilitation from the comfort of their homes. However, the small number of identified studies indicates that further research is warranted, both for the development of new technologies, as well as the potential health and psychosocial benefits for the PD population.

## Supporting information

S1 Appendix PRISMA-ScR checklist. (PDF)

S2 Appendix Search terms and search strategies. (DOCX)

## References

[1] Postuma RB, Berg D, Stern M, Poewe W, Olanow CW, Oertel W, et al. MDS clinical diagnostic criteria for Parkinson’s disease. Mov Disord. 2015;30(12):1591–601. doi: 10.1002/mds.26424 26474316

[2] Collaborators GBDP s D. Global, regional, and national burden of Parkinson’s disease, 1990-2016: a systematic analysis for the Global Burden of Disease Study 2016. Lancet Neurol. 2018;17(11):939–53. 30287051 10.1016/S1474-4422(18)30295-3 PMC6191528

[3] van Laar T, Chaudhuri KR, Antonini A, Henriksen T, Trošt M. Infusion therapies in the treatment of Parkinson’s disease. J Parkinsons Dis. 2023;13(5):641–57. doi: 10.3233/JPD-225112 37334617 PMC10473148

[4] Kashif M, Ahmad A, Mohseni Bandpei MA, Gillani SA. The combined effects of virtual reality with motor imagery techniques in patients with Parkinson’s disease. J Pak Med Assoc. 2022;72(12):2549–54. doi: 10.47391/JPMA.4856 37246689

[5] Yang Y, Wang Y, Gao T, Reyila A, Liu J, Liu J, et al. Effect of physiotherapy interventions on motor symptoms in people with Parkinson’s disease: A systematic review and meta-analysis. Biol Res Nurs. 2023;25(4):586–605. doi: 10.1177/10998004231171587 37070664

[6] Ernst M, Folkerts A-K, Gollan R, Lieker E, Caro-Valenzuela J, Adams A, et al. Physical exercise for people with Parkinson’s disease: a systematic review and network meta-analysis. Cochrane Database Syst Rev. 2023;1(1):CD013856. doi: 10.1002/14651858.CD013856.pub2 36602886 PMC9815433

[7] Jack K, McLean SM, Moffett JK, Gardiner E. Barriers to treatment adherence in physiotherapy outpatient clinics: a systematic review. Man Ther. 2010;15(3):220–8. doi: 10.1016/j.math.2009.12.004 20163979 PMC2923776

[8] Sluijs EM, Kok GJ, van der Zee J. Correlates of exercise compliance in physical therapy. Phys Ther. 1993;73(11):771–82; discussion 783-6. doi: 10.1093/ptj/73.11.771 8234458

[9] Reilly K, Lovejoy B, Williams R, Roth H. Differences between a supervised and independent strength and conditioning program with chronic low back syndromes. J Occup Med. 1989;31(6):547–50. doi: 10.1097/00043764-198906000-00012 2525182

[10] Nelson BW, O’Reilly E, Miller M, Hogan M, Wegner JA, Kelly C. The clinical effects of intensive, specific exercise on chronic low back pain: a controlled study of 895 consecutive patients with 1-year follow up. Orthopedics. 1995;18(10):971–81. doi: 10.3928/0147-7447-19951001-05 8584467

[11] Özden F, Sarı Z, Karaman ÖN, Aydoğmuş H. The effect of video exercise-based telerehabilitation on clinical outcomes, expectation, satisfaction, and motivation in patients with chronic low back pain. Ir J Med Sci. 2022;191(3):1229–39. doi: 10.1007/s11845-021-02727-8 34357527

[12] Agostini M, Moja L, Banzi R, Pistotti V, Tonin P, Venneri A, et al. Telerehabilitation and recovery of motor function: a systematic review and meta-analysis. J Telemed Telecare. 2015;21(4):202–13. doi: 10.1177/1357633X15572201 25712109

[13] Lange B, Flynn SM, Rizzo AA. Game-based telerehabilitation. Eur J Phys Rehabil Med. 2009;45(1):143–51. 19282807

[14] Sailer M, Hense JU, Mayr SK, Mandl H. How gamification motivates: An experimental study of the effects of specific game design elements on psychological need satisfaction. Computers in Human Behavior. 2017;69:371–80.

[15] Lina C, Guoen C, Huidan W, Yingqing W, Ying C, Xiaochun C, et al. The Effect of Virtual Reality on the Ability to Perform Activities of Daily Living, Balance During Gait, and Motor Function in Parkinson Disease Patients: A Systematic Review and Meta-Analysis. Am J Phys Med Rehabil. 2020;99(10):917–24. doi: 10.1097/PHM.0000000000001447 32304383

[16] Canning CG, Allen NE, Nackaerts E, Paul SS, Nieuwboer A, Gilat M. Virtual reality in research and rehabilitation of gait and balance in Parkinson disease. Nat Rev Neurol. 2020;16(8):409–25. doi: 10.1038/s41582-020-0370-2 32591756

[17] Lahude AB, Souza Corrêa P, P Cabeleira ME, Cechetti F. The impact of virtual reality on manual dexterity of Parkinson’s disease subjects: a systematic review. Disabil Rehabil Assist Technol. 2023;18(7):1237–44. doi: 10.1080/17483107.2021.2001060 35077662

[18] Truijen S, Abdullahi A, Bijsterbosch D, van Zoest E, Conijn M, Wang Y, et al. Effect of home-based virtual reality training and telerehabilitation on balance in individuals with Parkinson disease, multiple sclerosis, and stroke: a systematic review and meta-analysis. Neurol Sci. 2022;43(5):2995–3006. doi: 10.1007/s10072-021-05855-2 35175439 PMC9023738

[19] Zhu S, Sui Y, Shen Y, Zhu Y, Ali N, Guo C, et al. Effects of Virtual Reality Intervention on Cognition and Motor Function in Older Adults With Mild Cognitive Impairment or Dementia: A Systematic Review and Meta-Analysis. Front Aging Neurosci. 2021;13:586999. doi: 10.3389/fnagi.2021.586999 34025384 PMC8136286

[20] Arksey H, O’Malley L. Scoping studies: towards a methodological framework. Int J Soc Res Methodol. 2005;8(1):19–32.

[21] Levac D, Colquhoun H, O’Brien KK. Scoping studies: advancing the methodology. Implement Sci. 2010;5:69. doi: 10.1186/1748-5908-5-69 20854677 PMC2954944

[22] Tricco AC, Lillie E, Zarin W, O’Brien KK, Colquhoun H, Levac D, et al. PRISMA Extension for Scoping Reviews (PRISMA-ScR): Checklist and Explanation. Ann Intern Med. 2018;169(7):467–73. doi: 10.7326/M18-0850 30178033

[23] Covidence systematic review software. Melbourne, Australia: Veritas Health Innovation. n.d.

[24] Downs SH, Black N. The feasibility of creating a checklist for the assessment of the methodological quality both of randomised and non-randomised studies of health care interventions. J Epidemiol Community Health. 1998;52(6):377–84. doi: 10.1136/jech.52.6.377 9764259 PMC1756728

[25] Burns PB, Rohrich RJ, Chung KC. The levels of evidence and their role in evidence-based medicine. Plast Reconstr Surg. 2011;128(1):305–10. doi: 10.1097/PRS.0b013e318219c171 21701348 PMC3124652

[26] Albiol-Pérez S, Gil-Gómez J-A, Muñoz-Tomás M-T, Gil-Gómez H, Vial-Escolano R, Lozano-Quilis J-A. The Effect of Balance Training on Postural Control in Patients with Parkinson’s Disease Using a Virtual Rehabilitation System. Methods Inf Med. 2017;56(2):138–44. doi: 10.3414/ME16-02-0004 28244545

[27] Amprimo G, Masi G, Priano L, Azzaro C, Galli F, Pettiti G, et al. Assessment Tasks and Virtual Exergames for Remote Monitoring of Parkinson’s Disease: An Integrated Approach Based on Azure Kinect. Sensors (Basel). 2022;22(21):8173. doi: 10.3390/s22218173 36365870 PMC9654712

[28] Cikajlo I, et al. Telerehabilitation of upper extremities with target based games for persons with Parkinson’s disease. In: International Conference on Virtual Rehabilitation (CVR), 2017. 1–2.

[29] Dauvergne C, Bégel V, Gény C, Puyjarinet F, Laffont I, Dalla Bella S. Home-based training of rhythmic skills with a serious game in Parkinson’s disease: Usability and acceptability. Ann Phys Rehabil Med. 2018;61(6):380–5. doi: 10.1016/j.rehab.2018.08.002 30193992

[30] Nuic D, Vinti M, Karachi C, Foulon P, Van Hamme A, Welter M-L. The feasibility and positive effects of a customised videogame rehabilitation programme for freezing of gait and falls in Parkinson’s disease patients: a pilot study. J Neuroeng Rehabil. 2018;15(1):31. doi: 10.1186/s12984-018-0375-x 29636105 PMC5894136

[31] Palacios-Navarro G, Garcia-Magarino I, Ramos-Lorente P. A Kinect-based system for lower limb rehabilitation in Parkinson’s disease patients: a pilot study. J Med Syst. 2015;39(9):103. 26265237 10.1007/s10916-015-0289-0

[32] Pelosin E, et al. A game-console to improve balance in Parkinson disease: preliminary results using the Nintendo Wii. Italian Journal of Physiotherapy. 2012;2(2):45–9.

[33] Bacha JMR, et al. Effects of virtual rehabilitation on postural control of individuals with Parkinson disease. Motricidade. 2021;17:220–7.

[34] Blanc M, Roy A-L, Fraudet B, Piette P, Le Toullec E, Nicolas B, et al. Evaluation of a Digitally Guided Self-Rehabilitation Device Coupled With Telerehabilitation Monitoring in Patients With Parkinson Disease (TELEP@RK): Open, Prospective Observational Study. JMIR Serious Games. 2022;10(1):e24946. doi: 10.2196/24946 35129449 PMC8861867

[35] Brachman A, Marszałek W, Kamieniarz A, Michalska J, Pawłowski M, Juras G, Biomechanical measures of balance after balance-based exergaming training dedicated for patients with Parkinson’s disease. Gait Posture. 2021;87:170–6. doi: 10.1016/j.gaitpost.2021.04.036 33940308

[36] Cikajlo I, Hukić A, Dolinšek I, Zajc D, Vesel M, Krizmanič T, et al. Can telerehabilitation games lead to functional improvement of upper extremities in individuals with Parkinson’s disease?. Int J Rehabil Res. 2018;41(3):230–8. doi: 10.1097/MRR.0000000000000291 29757774 PMC6092088

[37] Cikajlo I, Hukić A, Zajc D. Exergaming as Part of the Telerehabilitation Can Be Adequate to the Outpatient Training: Preliminary Findings of a Non-randomized Pilot Study in Parkinson’s Disease. Front Neurol. 2021;12:625225. doi: 10.3389/fneur.2021.625225 33815252 PMC8010686

[38] Cornejo Thumm P, Giladi N, Hausdorff JM, Mirelman A. Tele-Rehabilitation with Virtual Reality: A Case Report on the Simultaneous, Remote Training of Two Patients with Parkinson Disease. Am J Phys Med Rehabil. 2021;100(5):435–8. doi: 10.1097/PHM.0000000000001745 33819924

[39] Esculier J-F, Vaudrin J, Bériault P, Gagnon K, Tremblay LE. Home-based balance training programme using Wii Fit with balance board for Parkinsons’s disease: a pilot study. J Rehabil Med. 2012;44(2):144–50. doi: 10.2340/16501977-0922 22266676

[40] Hashemi Y, Taghizadeh G, Azad A, Behzadipour S. The effects of supervised and non-supervised upper limb virtual reality exercises on upper limb sensory-motor functions in patients with idiopathic Parkinson’s disease. Hum Mov Sci. 2022;85:102977. doi: 10.1016/j.humov.2022.102977 35932518

[41] Herz NB, Mehta SH, Sethi KD, Jackson P, Hall P, Morgan JC. Nintendo Wii rehabilitation (“Wii-hab”) provides benefits in Parkinson’s disease. Parkinsonism Relat Disord. 2013;19(11):1039–42. doi: 10.1016/j.parkreldis.2013.07.014 23968649

[42] Holmes JD. The effects of a home-based virtual reality rehabilitation program on balance among individuals with Parkinson’s disease. Physical & Occupational Therapy In Geriatrics. 2013;31(3):241–53.

[43] Vallabhajosula S, McMillion AK, Freund JE. The effects of exergaming and treadmill training on gait, balance, and cognition in a person with Parkinson’s disease: A case study. Physiother Theory Pract. 2017;33(12):920–31. doi: 10.1080/09593985.2017.1359867 28812419

[44] Zeigelboim BS, José MR, Severiano MIR, Santos GJBD, Teive HAG, Liberalesso PBN, et al. The Use of Exergames in the Neurorehabilitation of People with Parkinson Disease: The Impact on Daily Life. Int Arch Otorhinolaryngol. 2021;25(1):e64–70. doi: 10.1055/s-0040-1702973 33542753 PMC7850887

[45] Barth M, Möbius R, Themann P, Güresir E, Matzke C, Winkler D, et al. Functional improvement of patients with Parkinson syndromes using a rehabilitation training software. Front Neurol. 2023;14:1210926. doi: 10.3389/fneur.2023.1210926 37645604 PMC10461806

[46] Gandolfi M, Geroin C, Dimitrova E, Boldrini P, Waldner A, Bonadiman S, et al. Virtual Reality Telerehabilitation for Postural Instability in Parkinson’s Disease: A Multicenter, Single-Blind, Randomized, Controlled Trial. Biomed Res Int. 2017;2017:7962826. doi: 10.1155/2017/7962826 29333454 PMC5733154

[47] Negrini S, et al. Nintendo Wii Fit for balance rehabilitation in patients with Parkinson’s disease: A comparative study. J Bodyw Mov Ther. 2017;21(1):117–23. 28167167 10.1016/j.jbmt.2016.06.001

[48] Nuic D, et al. Home-based exergaming to treat gait and balance disorders in patients with Parkinson’s disease: A phase II randomized controlled trial. Eur J Neurol. 2024;31(1):e16055. 10.1111/ene.16055 PMC11236010 37691341

[49] Del Pino R, de Echevarría AO, Díez-Cirarda M, Ustarroz-Aguirre I, Caprino M, Liu J, et al. Virtual coach and telerehabilitation for Parkinson´s disease patients: vCare system. J Public Health (Berl). 2023;33(7):1583–96. doi: 10.1007/s10389-023-02082-1

[50] Hoehn MM, Yahr MD. Parkinsonism: onset, progression and mortality. Neurology. 1967;17(5):427–42. doi: 10.1212/wnl.17.5.427 6067254

[51] Goetz CG, Tilley BC, Shaftman SR, Stebbins GT, Fahn S, Martinez-Martin P, et al. Movement Disorder Society-sponsored revision of the Unified Parkinson’s Disease Rating Scale (MDS-UPDRS): scale presentation and clinimetric testing results. Mov Disord. 2008;23(15):2129–70. doi: 10.1002/mds.22340 19025984

[52] Folstein MF, Folstein SE, McHugh PR. “Mini-mental state”. A practical method for grading the cognitive state of patients for the clinician. J Psychiatr Res. 1975;12(3):189–98. doi: 10.1016/0022-3956(75)90026-6 1202204

[53] Nasreddine ZS, Phillips NA, Bédirian V, Charbonneau S, Whitehead V, Collin I, et al. The Montreal Cognitive Assessment, MoCA: a brief screening tool for mild cognitive impairment. J Am Geriatr Soc. 2005;53(4):695–9. doi: 10.1111/j.1532-5415.2005.53221.x 15817019

[54] Berg KO, Wood-Dauphinee SL, Williams JI, Maki B. Measuring balance in the elderly: validation of an instrument. Can J Public Health. 1992;83 Suppl 2:S7-11. 1468055

[55] Tinetti ME. Performance-oriented assessment of mobility problems in elderly patients. J Am Geriatr Soc. 1986;34(2):119–26. doi: 10.1111/j.1532-5415.1986.tb05480.x 3944402

[56] Myers AM, et al. Discriminative and evaluative properties of the activities-specific balance confidence (ABC) scale. J Gerontol A Biol Sci Med Sci. 1998;53(4):M287-94. 10.1093/gerona/53a.4.m287 18314568

[57] Venkatesh V, Morris MG, Davis GB, Davis FD. User acceptance of information technology: Toward a unified view. MIS Quarterly. 2003;27(3):425–78.

[58] Mathiowetz V, Volland G, Kashman N, Weber K. Adult norms for the Box and Block Test of manual dexterity. Am J Occup Ther. 1985;39(6):386–91. doi: 10.5014/ajot.39.6.386 3160243

[59] Oxford Grice K, Vogel KA, Le V, Mitchell A, Muniz S, Vollmer MA. Adult norms for a commercially available Nine Hole Peg Test for finger dexterity. Am J Occup Ther. 2003;57(5):570–3. doi: 10.5014/ajot.57.5.570 14527120

[60] Jebsen RH, Taylor N, Trieschmann RB, Trotter MJ, Howard LA. An objective and standardized test of hand function. Arch Phys Med Rehabil. 1969;50(6):311–9. 5788487

[61] Ellis TD, Colón-Semenza C, DeAngelis TR, Thomas CA, Hilaire M-HS, Earhart GM, et al. Evidence for Early and Regular Physical Therapy and Exercise in Parkinson’s Disease. Semin Neurol. 2021;41(2):189–205. doi: 10.1055/s-0041-1725133 33742432 PMC8678920

[62] Zaman MS, Ghahari S, McColl MA. Barriers to Accessing Healthcare Services for People with Parkinson’s Disease: A Scoping Review. J Parkinsons Dis. 2021;11(4):1537–53. doi: 10.3233/JPD-212735 34308913 PMC8609702

[63] Foundation PS. Parkinson’s Foundation Exercise Recommendations. 2021.

[64] Duncan RP, Earhart GM. Measuring participation in individuals with Parkinson disease: relationships with disease severity, quality of life, and mobility. Disabil Rehabil. 2011;33(15–16):1440–6. doi: 10.3109/09638288.2010.533245 21091047

[65] Asgharzadeh Chamleh MR, Afkanpour M, Tehrany Dehkordy D, Norouzkhani N, Aalaei S. Game-based telerehabilitation in neurological disorders: a systematic review of features, opportunities and challenges. Disabil Rehabil Assist Technol. 2025;20(5):1257–71. doi: 10.1080/17483107.2025.2450010 39817668

[66] Vellata C, Belli S, Balsamo F, Giordano A, Colombo R, Maggioni G. Effectiveness of Telerehabilitation on Motor Impairments, Non-motor Symptoms and Compliance in Patients With Parkinson’s Disease: A Systematic Review. Front Neurol. 2021;12:627999. doi: 10.3389/fneur.2021.627999 34512495 PMC8427282

[67] Aminov A, Rogers JM, Middleton S, Caeyenberghs K, Wilson PH. What do randomized controlled trials say about virtual rehabilitation in stroke? A systematic literature review and meta-analysis of upper-limb and cognitive outcomes. J Neuroeng Rehabil. 2018;15(1):29. doi: 10.1186/s12984-018-0370-2 29587853 PMC5870176

[68] Matamala-Gomez M, Maisto M, Montana JI, Mavrodiev PA, Baglio F, Rossetto F, et al. The Role of Engagement in Teleneurorehabilitation: A Systematic Review. Front Neurol. 2020;11:354. doi: 10.3389/fneur.2020.00354 32435227 PMC7218051

