# Supplementary material for: Gamification-based tele-rehabilitation for physical therapy in patients with Parkinson’s disease: A scoping review
Source: PLoS One. 2025 Aug 29;20(8):e0326705. doi: 10.1371/journal.pone.0326705 (PMC12396673; doi:10.1371/journal.pone.0326705)
Supplement: S2 Appendix — (DOCX) [file pone.0326705.s002.docx]

# Supplementary Material 2: Search Terms and Search Strategies

## I.1. OVID MEDLINE

|  |  |  |
| --- | --- | --- |
| 1 | software/ or gamification/ or mobile applications/ or software design/ or user-centered design/ or exp user-computer interface/ or exp video games/ | 170068 |
| 2 | ((mobile* or cellphone* or cell phone* or iOS* or android*) adj4 (app* or program*)).tw,kf. | 16349 |
| 3 | (Game* or gamifi* or software* or user-centered design* or user centered design* or user centred design* or user-centred design* or exergaming* or wii* or user-computer interface* or user computer interface* or human machine interface* or human-machine interface*).tw,kf. | 288942 |
| 4 | or/1-3 | 416014 |
| 5 | telerehabilitation/ | 840 |
| 6 | (((tele* or Remote* or Virtual* or home* or Unsupervise*) adj4 (((exercis* or physical* or dance*) adj4 therapy*) or physiotherap*)) or e-exercis* or e exercise* or e-physical therapy* or e physical therapy* or e-dance therapy* or e dance therapy* or edance therapy* or ephysiotherap* e physiotherap* or e-physiotherap*).tw,kf. | 1356 |
| 7 | (((tele* or Remote* or Virtual* or home* or Unsupervise*) adj4 rehab*) or telerehab* or erehab* or e-rehab* or e rehab*).tw,kf. | 6533 |
| 8 | (((tele* or Remote* or Virtual* or home* or Unsupervise*) adj4 ((physical* or physiotherap* musculoskeletal* or myofunctional* or enduranc* or muscle* stretch* or plyometric* or resistanc*) adj4 (exercis* or activity* or activit* or movement* or training*))) or ((ephysical* or e physical* or e-physical* or ephysiotherap* or e-physiotherap* or e physiotherap* or emusculoskeletal* or e musculoskeletal* or e-musculoskeletal* or emyofunctional* or e-myofunctional* or e myofunctional* or enduranc* or e enduranc* or e-enduranc*or e muscle* stretch* or e-muscle* stretch* or emuscle* stretch* or eplyometric* or e plyometric* or e-plyometric* or eresistacn* or e-resistacn* or e resistacn*) adj4 (exercis* or activity* or activit* or movement* or training*))).tw,kf. | 15982 |
| 9 | or/5-8 | 23571 |
| 10 | 4 and 9 | 1342 |
| 11 | limit 10 to (english language and yr="2010 -Current") | 1129 |

## I.2. OVID EMBASE

| 1 | exp software/ | 287522 |
| --- | --- | --- |
| 2 | exp human machine interface/ | 45245 |
| 3 | exp game/ | 8693 |
| 4 | exp video game/ | 5474 |
| 5 | ((mobile* or cellphone* or cell phone* or iOS* or android*) adj4 (app* or program*)).tw,kf. | 20616 |
| 6 | (Game* or gamifi* or software* or user-centered design* or user centered design* or user centred design* or user-centred design* or exergaming* or wii* or user-computer interface* or user computer interface* or human machine interface* or human-machine interface*).tw,kf. | 446571 |
| 7 | or/1-6 | 656104 |
| 8 | exp telerehabilitation/ | 1950 |
| 9 | (((tele* or Remote* or Virtual* or home* or Unsupervise*) adj4 (((exercis* or physical* or dance*) adj4 therapy*) or physiotherap*)) or e-exercis* or e exercise* or e-physical therapy* or e physical therapy* or e-dance therapy* or e dance therapy* or edance therapy* or ephysiotherap* e physiotherap* or e-physiotherap*).tw,kf. | 2289 |
| 10 | (((tele* or Remote* or Virtual* or home* or Unsupervise*) adj4 rehab*) or telerehab* or erehab* or e-rehab* or e rehab*).tw,kf. | 10007 |
| 11 | (((tele* or Remote* or Virtual* or home* or Unsupervise*) adj4 ((physical* or physiotherap* musculoskeletal* or myofunctional* or enduranc* or muscle* stretch* or plyometric* or resistanc*) adj4 (exercis* or activity* or activit* or movement* or training*))) or ((ephysical* or e physical* or e-physical* or ephysiotherap* or e-physiotherap* or e physiotherap* or emusculoskeletal* or e musculoskeletal* or e-musculoskeletal* or emyofunctional* or e-myofunctional* or e myofunctional* or enduranc* or e enduranc* or e-enduranc*or e muscle* stretch* or e-muscle* stretch* or emuscle* stretch* or eplyometric* or e plyometric* or e-plyometric* or eresistacn* or e-resistacn* or e resistacn*) adj4 (exercis* or activity* or activit* or movement* or training*))).tw,kf. | 21029 |
| 12 | or/8-11 | 32994 |
| 13 | 7 and 12 | 2076 |
| 14 | limit 13 to (english language and yr="2010 -Current") | 1826 |
| 15 | limit 14 to (article or article in press or books or chapter or conference abstract or conference paper or "conference review" or editorial or erratum or letter) | 1639 |

## I.3. [CINAHL Plus](http://myaccess.library.utoronto.ca/login?url=http://search.ebscohost.com/login.aspx?authtype=ip,uid&custid=s3409242&profile=ehost&defaultdb=cin20) (Nursing & allied health sciences)

| # | Query | Results |
| --- | --- | --- |
| S1 | (MH "Software+") OR (MH "User-Computer Interface+") | 448,234 |
| S2 | (MH "Software Design") | 4,195 |
| S3 | (MH "User-Computer Interface+") | 12,172 |
| S4 | (MH "Games+") | 11,186 |
| S5 | (MH "Video Games+") | 5,655 |
| S6 | TI ( ((mobile* or cellphone* or cell phone* or iOS* or android*) N4 (app* or program*)) ) OR AB ( ((mobile* or cellphone* or cell phone* or iOS* or android*) N4 (app* or program*)) ) | 7,810 |
| S7 | TI ( Game* or gamifi* or software* or user-centered design* or user centered design* or user centred design* or user-centred design* or exergaming* or wii* or user-computer interface* or user computer interface* or human machine interface* or human-machine interface* ) OR AB ( Game* or gamifi* or software* or user-centered design* or user centered design* or user centred design* or user-centred design* or exergaming* or wii* or user-computer interface* or user computer interface* or human machine interface* or human-machine interface* ) | 83,412 |
| S8 | S1 OR S2 OR S3 OR S4 OR S5 OR S6 OR S7 | 515,986 |
| S9 | (MH "Telerehabilitation") | 506 |
| S10 | TI ( (((tele* or Remote* or Virtual* or home* or Unsupervise*) N4 (((exercis* or physical* or dance*) N4 therapy*) or physiotherap*)) or e-exercis* or e exercise* or e-physical therapy* or e physical therapy* or e-dance therapy* or e dance therapy* or edance therapy* or ephysiotherap* e physiotherap* or e-physiotherap*) ) OR AB ( (((tele* or Remote* or Virtual* or home* or Unsupervise*) N4 (((exercis* or physical* or dance*) N4 therapy*) or physiotherap*)) or e-exercis* or e exercise* or e-physical therapy* or e physical therapy* or e-dance therapy* or e dance therapy* or edance therapy* or ephysiotherap* e physiotherap* or e-physiotherap*) ) | 1,256 |
| S11 | TI ( (((tele* or Remote* or Virtual* or home* or Unsupervise*) N4 rehab*) or telerehab* or erehab* or e-rehab* or e rehab*) ) OR AB ( (((tele* or Remote* or Virtual* or home* or Unsupervise*) N4 rehab*) or telerehab* or erehab* or e-rehab* or e rehab*) ) | 4,628 |
| S12 | TI ( (((tele* or Remote* or Virtual* or home* or Unsupervise*) N4 ((physical* or physiotherap* musculoskeletal* or myofunctional* or enduranc* or muscle* stretch* or plyometric* or resistanc*) N4 (exercis* or activity* or activit* or movement* or training*))) or ((ephysical* or e physical* or e-physical* or ephysiotherap* or e-physiotherap* or e physiotherap* or emusculoskeletal* or e musculoskeletal* or e-musculoskeletal* or emyofunctional* or e-myofunctional* or e myofunctional* or enduranc* or e enduranc* or e-enduranc*or e muscle* stretch* or e-muscle* stretch* or emuscle* stretch* or eplyometric* or e plyometric* or e-plyometric* or eresistacn* or e-resistacn* or e resistacn*) N4 (exercis* or activity* or activit* or movement* or training*))) ) OR AB ( (((tele* or Remote* or Virtual* or home* or Unsupervise*) N4 ((physical* or physiotherap* musculoskeletal* or myofunctional* or enduranc* or muscle* stretch* or plyometric* or resistanc*) N4 (exercis* or activity* or activit* or movement* or training*))) or ((ephysical* or e physical* or e-physical* or ephysiotherap* or e-physiotherap* or e physiotherap* or emusculoskeletal* or e musculoskeletal* or e-musculoskeletal* or emyofunctional* or e-myofunctional* or e myofunctional* or enduranc* or e enduranc* or e-enduranc*or e muscle* stretch* or e-muscle* stretch* or emuscle* stretch* or eplyometric* or e plyometric* or e-plyometric* or eresistacn* or e-resistacn* or e resistacn*) N4 (exercis* or activity* or activit* or movement* or training*)))) | 6,766 |
| S13 | S9 OR S10 OR S11 OR S12 | 12,457 |
| S14 | S8 AND S13 | 2,501 |
| S15 | S8 AND S13: Limiters - Published Date: 20100101-20221231 | 1,878 |

## I.4. Scopus

| 1 | TITLE-ABS-KEY ( ( ( tele* OR remote* OR virtual* OR home* OR unsupervis* ) W/4 ( ( ( exercis* OR physical* OR dance* ) W/4 therapy* ) OR physiotherap* ) ) ) | 2,333 |
| --- | --- | --- |
| 2 | TITLE-ABS-KEY ( ( "e exercise*" OR "e physical therapy*" OR "ephysical therapy*" OR "e dance therapy*" OR "edance therapy*" OR "e physiotherap*" OR "ephysiotherap*" ) ) | 361 |
| 3 | TITLE-ABS-KEY ( ( ( tele* OR remote* OR virtual* OR home* OR unsupervis* ) W/4 ( physical* OR physiotherap* OR musculoskeletal* OR myofunction* OR endurance* OR muscle* OR stretch OR plyometric* OR resistanc* ) W/4 ( exercis* OR activit?* OR training* OR movement* ) ) ) | 4,749 |
| 4 | TITLE-ABS-KEY ( ( ( ephysical* OR "e physical*" OR ephysiotherap* OR "e physiotherap*" OR emusculoskeletal* OR "e musculoskeletal*" OR emyofunctional* OR "e myofunctional*" OR enduranc* OR "e enduranc*" OR "e muscle*" OR estretch* OR "e stretch*" OR eplyometric* OR "e plyometric*" OR eresistacn* OR "e resistacn*" ) W/4 ( exercis* OR activit* OR movement* OR training* ) ) ) | 21,836 |
| 5 | TITLE-ABS-KEY ( ( ( tele* OR remote* OR virtual* OR home* OR unsupervis* ) W/4 rehab* ) ) | 10,553 |
| 6 | TITLE-ABS-KEY ( ( ( ( tele* OR remote* OR virtual* OR home* OR unsupervise* ) W/4 rehab* ) OR telerehab* OR erehab* "e rehab*" ) ) | 23 |
| 7 | 1 OR 2 OR 3 OR 4 OR 5 OR 6 | 38,645 |
| 8 | TITLE-ABS-KEY ( game* OR gamif?* OR software OR "user cent??ed design" OR exergaming* OR wii* OR "user computer interface*" OR "human machine interface*" ) | 2,115,618 |
| 9 | TITLE-ABS-KEY ( ( ( mobile* OR cellphone* OR "cell phone*" OR ios* OR android* ) W/4 ( application* OR app* OR program* ) ) ) | 141,325 |
| 10 | 8 OR 9 | 2,232,634 |
| 11 | 7 AND 10 | 2,924 |
| 12 | LIMIT 11 to 2010-current and exclude conference papers | 1696 |

Total number of articles after de-duplication: 4078 articles
